# Electromagnetism in Linear, Homogeneous and Isotropic Materials: The Analogy Between Electricity and Magnetism in the Susceptibility and Polarization

**DOI:** 10.3390/ma18184282

**Published:** 2025-09-12

**Authors:** Dimosthenis Stamopoulos

**Affiliations:** Department of Physics, School of Science, National and Kapodistrian University of Athens, Zografou Panepistimioupolis, 15784 Athens, Greece; densta@phys.uoa.gr

**Keywords:** electromagnetism, electric susceptibility, electric polarization, dielectrics, extrinsic susceptibility, demagnetizing effect, demagnetizing factor

## Abstract

Through the years, the asymmetry in the constitutive relations that define the electric and magnetic polarization, **P** and **M**, respectively, by the relevant vector field, **E** and **H**, has been imprinted, rather arbitrarily, in Maxwell’s equations. Accordingly, in linear, homogeneous, and isotropic (LHI) materials, the electric and magnetic polarization are defined via **P** = χ_e_ε_0_**E** (‘P-E, χ_e_’ formulation; 0 ≤ χ_e_ < ∞) and **M** = χ_m_**H** (‘M-H, χ_m_’ formulation; −1 ≤ χ_m_ < ∞), respectively. Recently, the constitutive relation of the polarization was revisited in LHI dielectrics by introducing an electric susceptibility, χ_ε_, which couples linearly the reverse polarization, $\tilde{P}$ = −**P**, with the electric displacement **D** through $\tilde{P}$ = χ_ε_**D** (‘P-D, χ_ε_’ formulation; −1 ≤ χ_ε_ ≤ 0). Here, the ‘P-D, χ_ε_’ formulation is generalized for the time-dependent case. It is documented that the susceptibility and polarization of LHI dielectric and magnetic materials can be described by the ‘P-D, χ_ε_’ and ‘M-H, χ_m_’ formulation, respectively, on a common basis. To this end, the depolarizing effect is taken into account, which unavoidably emerges in realistic specimens of limited size, by introducing a series scheme to describe the evolution of polarization and calculate the *extrinsic* susceptibility. The engagement of the depolarizing factor N (0 ≤ N≤ 1) with the accompanying convergence conditions dictates that the *intrinsic* susceptibility of LHI materials, whether electric or magnetic, should range within [−1, 1]. The ‘P-D, χ_ε_’ and ‘M-H, χ_m_’ formulations conform with this expectation, while the ‘P-E, χ_e_’ does not. Remarkably, Maxwell’s equations are unaltered by the ‘P-D, χ_ε_’ formulation. Thus, all time-dependent processes of electromagnetism described by the standard ‘P-E, χ_e_’ approach, are reproduced equivalently, or even advantageously, by the alternative ‘P-D, χ_ε_’ formulation.

## 1. Introduction

Through the decades, electromagnetism [1,2,3] has attained the well-established form mathematically represented by Maxwell’s equations [4,5,6,7,8,9,10,11,12,13,14,15].

(1) $$\nabla \cdot E\left(r,t\right)=\rho (r,t)/\varepsilon_{0}$$

(2) $$\nabla \cdot Β\left(r,t\right)=0$$

(3) $$\nabla \times E\left(r,t\right)=-\partial Β(r,t)/\partial t$$

(4) $$\nabla \times Β\left(r,t\right)=\mu_{0}\left(J\left(r,t\right)+\varepsilon_{0}\partial E\left(r,t\right)/\partial t\right).$$

Here, $E\left(r,t\right)$ and $Β\left(r,t\right)$ are the vector fields formally called electric and magnetic flux density, respectively, and $\rho \left(r,t\right)$ and $J\left(r,t\right)$ refer to the total (*free* and *bound*) density of charge and current, respectively, while $\varepsilon_{0}$ is the permittivity (8.854… × 10^−12^ F/m) and $\mu_{0}$ is the permeability (1.257… × 10^−6^ N/A^2^), of vacuum. Except for the electrical properties of conductors, which relate to *free* charges and currents, a key property of electromagnetism in matter is the polarization, electric and magnetic, a vector field, P(r,t) and M(r,t), respectively. Here, the term ‘polarization’ is adopted in both electricity and magnetism (discriminating between P(r,t) and M(r,t), when needed), since both cases refer, by definition, to the local density and direction of the respective dipoles hosted in a material. Polarization, electric and magnetic, can be either induced or permanent [8,9,10,11,12,13,14,15]. In both cases, *bound* charges and currents can be introduced mathematically through the polarization [16,17,18,19,20].

Except for the above ‘microscopic’ form of Maxwell’s equations, the following, so-called ‘macroscopic’ form is also employed, depending on the circumstances [4,5,6,7,8,9,10,11,12,13,14,15].

(5) $$\nabla \cdot D\left(r,t\right)=\rho_{f}\left(r,t\right)$$

(6) $$\nabla \cdot Β\left(r,t\right)=0$$

(7) $$\nabla \times E\left(r,t\right)=-\partial Β(r,t)/\partial t$$

(8) $$\nabla \times H\left(r,t\right)=J_{f}\left(r,t\right)+\partial D\left(r,t\right)/\partial t$$

where $D\left(r,t\right)$ and $H\left(r,t\right)$ are two *auxiliary* fields, formally called electric displacement and magnetic field strength, respectively, while $\rho_{f}\left(r,t\right)$ and $J_{f}\left(r,t\right)$ refer to the relevant *free* density of charge and current.

Electromagnetism in the form employed today, accompanied by the constitutive relations for the definition of electric and magnetic polarization, exhibits some intriguing points that deserve attention.

The first intriguing point in the current electromagnetic theory is the obvious asymmetry in the constitutive relations which define the polarization, electric, P(r,t), and magnetic, M(r,t), even in the linear, homogeneous, and isotropic (LHI) materials discussed here. Curiously, through the years, this arbitrary asymmetry has been established as an inherent property of electromagnetism.

Specifically, the electric polarization, $P\left(r,t\right),$ is defined by means of E(r,t), which is most commonly considered as the *primary* field of electricity, through the relation [8,9,10,11,12,13,14,15].

(9) $$P(r,t)=\chi_{e}\varepsilon_{0}E(r,t).$$

Here, $\chi_{e}$ is the *intrinsic* electric susceptibility, a constant which depends on the endogenous properties of each LHI dielectric material (with $0\leq \chi_{e}<\infty$). For the sake of convenience, in the rest of the present work, this formulation is called ‘P-E, χ_e_’. Then, the so-called electric displacement, D(r,t), which is considered as the *secondary/auxiliary* field of electricity, is introduced through the constitutive relation [8,9,10,11,12,13,14,15].

(10) $$D(r,t)=\varepsilon_{0}E(r,t)+P(r,t).$$

The formulation of electricity used today states that E(r,t) relates to both *free* charges and the dielectric properties of the material (i.e., *bound* charges). The dependence of D(r,t) on both the *free* charges and the dielectric properties of the material is somehow obscured (most commonly, and quite erroneously, it is considered that D(r,t) depends only on *free* charges).

In contrast, the magnetic polarization, $M\left(r,t\right),$ is defined by means of H(r,t), which is most commonly considered as the *secondary/auxiliary* field of magnetism, through the relation [8,9,10,11,12,13,14,15].

(11) $$M\left(r,t\right)=\chi_{m}H\left(r,t\right).$$

Here, $\chi_{m}$ is the *intrinsic* magnetic susceptibility, a constant which depends on the endogenous properties of each LHI magnetic material (with $-1\leq \chi_{m}<\infty$). For the sake of convenience, in the rest of the present work, this formulation is called ‘M-H, χ_m_’. Then, the so-called magnetic flux density, B(r,t), which is considered as the *primary* field of magnetism, is introduced through the constitutive relation [8,9,10,11,12,13,14,15].

(12) $$B(r,t)=\mu_{0}\left(H(r,t)+M(r,t)\right).$$

The formulation of magnetism used today states that B(r,t) relates to both *free* currents and the magnetic properties of the material (i.e., *bound* currents). The dependence of the magnetic field, H(r,t), on both the *free* currents and the magnetic properties of the material is somehow obscured (most commonly, and quite erroneously, it is considered that H(r,t) depends exclusively on *free* currents).

Obviously, the complete analogy between electricity and magnetism requires that either P(r,t) should be defined through D(r,t) or M(r,t) should be defined through B(r,t).

A second notable point in the ‘P-E, χ_e_’ formulation of electricity relates to the rather misleading concept of the causality/feedback between the electric field, E(r,t), and the polarization, P(r,t). This has been discussed in detail in many textbooks. For instance, in [8] (pages 68 and 76), the causality between E(r,t) and $P\left(r,t\right)$ is described as an “*awkward situation*” since “*the polarization of the dielectric depends on the total electric field in the medium, but a part of the electric field is produced by the dielectric itself*”. Also, in [13] (page 186), the feedback between E(r,t) and $P\left(r,t\right)$ is described as an “*infinite regress*”. Details can be found in [21].

A third interesting point complicates the situation further and relates to a mathematical flaw that accompanies the misleading conceptual point discussed above. Specifically, in [21], representative realistic specimens were studied thoroughly, e.g., spheres and cylinders consisting of LHI parent dielectrics, by taking into account the depolarizing effect and the respective factor, N (with 0≤N≤1), which appear due to the specific shape and limited size of each specimen. Accordingly, the *extrinsic* susceptibility of the specimen was calculated with respect to the *intrinsic* susceptibility, $\chi_{e}$ (with $0\leq \chi_{e}<\infty$), of the parent LHI material. Importantly, in the representative cases studied in [21], it was clearly evidenced that the ‘P-E, χ_e_’ formulation of electricity suffers from its non-compliance with basic convergence conditions, $\left|-\chi_{e}N\right|<1$, when the physical process of the “*infinite regress*” between E(r) and $P\left(r\right)$ is simulated mathematically by means of series. Clearly, this mathematical flaw is inherent to the ‘P-E, χ_e_’ formulation of electricity due to the constitutive relation (9) used to define the relevant *intrinsic* susceptibility, $\chi_{e}$, and polarization, $P\left(r\right)$.

Finally, a fourth worthy point relates to the units-based preference that can be employed to define the constitutive relation between the polarization and the parent vector field in the LHI materials studied here. Briefly, in electricity, the constitutive relation between E(r), $D\left(r\right),$ and P(r) reads $D\left(r\right)=\varepsilon_{0}E\left(r\right)+P(r)$. This relation is universal, holds for all materials, and should not be disputed by any formulation. This relation highlights that, on physical grounds, the electric polarization, P(r), and the electric displacement, D(r), have the same units, and thus, the same physical content, in all materials (not only in the LHI ones studied here). On the contrary, P(r) and E(r) have different physical contents so that, except for the scalar dimensionless constant called electric susceptibility, $\chi_{e}$, when their connection is forced, adjustment of their units is needed. In the ‘P-E, χ_e_’ formulation (relation (9)), this is accomplished by introducing the vacuum permittivity, $\varepsilon_{0}$. On the contrary, in magnetism, i.e., in the so-called ‘M-H, χ_m_’ formulation, the constitutive relation between B(r), H(r), and M(r) reads $B\left(r\right)=\mu_{0}(H(r)+M(r))$. Now H(r) and M(r) have the same physical content in all materials, while B(r) matches through the incorporation of the vacuum permeability $\mu_{0}$. Thus, to define the magnetic polarization, M(r), in magnetism, it is well accepted to invoke the simplest choice, i.e., to employ the vector field H(r) through the introduction of a scalar dimensionless constant called magnetic susceptibility, $\chi_{m}$. By following the paradigm of magnetic polarization, in electricity, the same units-based preference calls us to relate the electric polarization, $P\left(r\right)$, directly to the electric displacement, $D\left(r\right)$, through a scalar dimensionless constant, since these two vector fields have the same physical content. Definitely, this is the simplest choice (in line with Occam’s razor). This was performed in [21] through the introduction of the so-called ‘P-D, χ_ε_’ formulation for the case of electrostatics.

Here, these issues are discussed for the time-dependent case of electromagnetism by following a direct and comparative approach between electricity and magnetism for the wide class of LHI materials. To this end, a new aspect is employed, the so-called ‘P-D, χ_ε_’ formulation, introduced recently in electrostatics [21]. In the ‘P-D, χ_ε_’ approach, the reverse polarization $\tilde{P}\left(r\right)=-P\left(r\right)$ couples linearly to the electric displacement, D(r), in a *direct* way, through the ‘P-D’ *intrinsic* electric susceptibility $\chi_{\varepsilon }$, with $-1\leq \chi_{\varepsilon }\leq 0$ [21]. Notice the difference in the subscript of the susceptibility, $\chi_{\varepsilon }$, of the ‘P-D, χ_ε_’ formulation, in comparison to the susceptibility, $\chi_{e}$, of the standard ‘P-E, χ_e_’ one. According to this aspect, in LHI materials, D(r) is the *primary* field, while $\tilde{P}\left(r\right)=-P\left(r\right)$ and $E\left(r\right)$ are *secondary* fields. The following linear relations hold between them [21]

(13) $$\tilde{P}\left(r\right)=-P\left(r\right)=\chi_{\varepsilon }D(r)$$

(14) $$E\left(r\right)=(1/\varepsilon_{\varepsilon })D\left(r\right)$$

(15) $$\tilde{P}\left(r\right)=-P\left(r\right)=(1-\varepsilon_{r\varepsilon })\varepsilon_{0}E\left(r\right)$$

where $\chi_{\varepsilon }$, $\varepsilon_{r\varepsilon },$ and $\varepsilon_{\varepsilon }$ refer to the ‘P-D’ *intrinsic* constants, that is, electric susceptibility, relative permittivity, and permittivity, respectively, which reflect the endogenous properties of the LHI dielectric material. These are defined through the relations [21]

(16) $$\varepsilon_{r\varepsilon }={\left(1+\chi_{\varepsilon }\right)}^{-1}$$

(17) $$\varepsilon_{\varepsilon }=\varepsilon_{0}\varepsilon_{r\varepsilon }$$

with $-1\leq \chi_{\varepsilon }\leq 0$ and $1\leq \varepsilon_{r\varepsilon }<\infty$. The ‘P-D’ electric susceptibility, relative permittivity, and permittivity relate to the ‘P-E’ ones through the relations

(18) $$1+\chi_{\varepsilon }=1/(1+\chi_{e})$$

(19) $$\varepsilon_{r\varepsilon }=\varepsilon_{r}$$

(20) $$\varepsilon_{\varepsilon }=\varepsilon .$$

Referring to the divergence of the three vector fields, we have [21]

(21) $$\nabla \cdot D\left(r\right)=\rho_{f}\left(r\right)$$

(22) $$\nabla \cdot \tilde{P}\left(r\right)=\nabla \cdot (-P\left(r\right))=\rho_{b}\left(r\right)$$

(23) $$\nabla \cdot E\left(r\right)=\rho \left(r\right)/\varepsilon_{0}.$$

Also, the linear coupling between the three vector fields $D\left(r\right)$, $\tilde{P}\left(r\right)=-P\left(r\right)$ and $E\left(r\right)$ is reflected onto the respective *free*, $\rho_{f}(r)$, *bound*, $\rho_{b}(r)$, and *total*, $\rho \left(r\right)=\rho_{f}\left(r\right)+\rho_{b}(r)$, charge densities, as [21]

(24) $$\rho_{b}\left(r\right)=\chi_{\varepsilon }\rho_{f}\left(r\right)$$

(25) $$\rho \left(r\right)=\left(1+\chi_{\varepsilon }\right)\rho_{f}\left(r\right)$$

(26) $$\rho \left(r\right)=\left(\left(1+\chi_{\varepsilon }\right)/\chi_{\varepsilon }\right)\rho_{b}\left(r\right)$$.

Finally, it is noted that once the reverse polarization $\tilde{P}\left(r\right)=-P\left(r\right)$ is known, the volume and surface density of the relevant reverse *bound* charges can be determined through the respective relations

(27) $${\tilde{\rho }}_{b}\left(r\right)=-\nabla \cdot \tilde{P}\left(r\right)=\nabla \cdot P\left(r\right)=-\rho_{b}\left(r\right)$$

(28) $${\tilde{\sigma }}_{b}\left(r\right){|}_{S}=\hat{n}\cdot \tilde{P}\left(r\right){|}_{S}=-\hat{n}\cdot P\left(r\right){|}_{S}={-\sigma }_{b}\left(r\right){|}_{S}.$$

In [21], the ‘P-D, χ_ε_’ formulation was introduced for the case of electrostatics. Here, the ‘P-D, χ_ε_’ formulation is exploited for the general, time-dependent case, in comparison to the standard ‘P-E, χ_e_’ and ‘M-H, χ_m_’ ones. It is shown that in LHI dielectric and magnetic materials, the susceptibility and polarization can be described on a common basis. Qualitatively, a schematic recursive sequence is employed to describe the physical processes of how the polarization evolves immediately after application of the external field, that is, at the transient stage, while it gradually obtains its final form at the steady state. Quantitatively, this process is investigated mathematically in a detailed and consistent way by means of series in all three formulations, the ‘P-E, χ_e_’, ‘M-H, χ_m_’, and ‘P-D, χ_ε_’. Notably, the depolarizing effect [22,23,24,25,26,27,28,29,30,31,32] is taken into account, which accompanies all realistic specimens of limited size through the respective depolarizing factors, $N_{e}$, $N_{m}$, and $N_{\varepsilon }$ (all three ranging within $0\leq N_{e},N_{m},N_{\varepsilon }\leq 1$) and calculate the *extrinsic* susceptibility of a spherical specimen in connection with the *intrinsic* susceptibility of the parent LHI material. Importantly, the engagement of the depolarizing factors $N_{e}$, $N_{m}$, and $N_{\varepsilon }$ sets limitations on the convergence of the series employed to describe the polarization; it emerges that in all three formulations, the following condition should hold: $\left|-\chi N\right|<1$ (where χ stands for each one of the *intrinsic* $\chi_{e}$, $\chi_{m}$, and $\chi_{\varepsilon }$, while N refers to the respective $N_{e}$, $N_{m}$, and $N_{\varepsilon }$). Given that $0\leq N_{e},N_{m},N_{\varepsilon }\leq 1$, for LHI materials, the *intrinsic* electric and magnetic susceptibility should range within $1\leq \chi_{e},\chi_{m},\chi_{\varepsilon }\leq 1$. When this condition is fulfilled, the convergence of the employed series is guaranteed. Interestingly, in the ‘P-E, χ_e_’ formulation of LHI dielectrics where $P(r,t)=\chi_{e}\varepsilon_{0}E(r,t)$ and $0\leq \chi_{e}<\infty$, the convergence condition, $\left|-\chi_{e}N_{e}\right|<1$, is not satisfied within the entire range $0\leq N_{e}\leq 1$. This is a strange drawback of the standard ‘P-E, χ_e_’ formulation. On the other hand, in the ‘M-H, χ_m_’ formulation of standard paramagnetic and diamagnetic LHI materials where $M\left(r,t\right)=\chi_{m}H\left(r,t\right)$ and $\chi_{m}$ ranges strictly within $1\leq \chi_{m}\leq 1$, the convergence condition, $\left|-\chi_{m}N_{m}\right|<1$, is fulfilled generically. This safe behavior of the ‘M-H, χ_m_’ formulation of magnetism is adopted in electricity through the ‘P-D, χ_ε_’ formulation where $\tilde{P}\left(r,t\right)=-P\left(r,t\right)=\chi_{\varepsilon }D\left(r,t\right)$ and $\chi_{\varepsilon }$ ranges within $1\leq \chi_{\varepsilon }\leq 0$. In this formulation of electricity, the respective convergence condition, $|-\chi_{\varepsilon }N_{\varepsilon }|<1$, is fulfilled generically, as well. These considerations document that, at least in LHI materials, the electric susceptibility and polarization can be reconceptualized on a basis completely analogous to that of magnetism. On the other hand, the equivalence of the ‘P-E, χ_e_’ and ‘P-D, χ_ε_’ formulations in the description of electricity is documented for the generic case. Interestingly, the complementary character of the depolarizing factors in the ‘P-E, χ_e_’ and ‘P-D, χ_ε_’ formulations is proved through the generic condition $N_{e}+N_{\varepsilon }=1$.

Finally, an important property of the ‘P-D, χ_ε_’ formulation is documented; it does not alter Maxwell’s equations. Thus, all relevant time-dependent processes/entities (wave equation, propagation and the respective characteristics, energy density, scattering, etc.) can be described by the ‘P-D, χ_ε_’ formulation in a way absolutely consistent with standard knowledge, as it should.

## 2. Susceptibility and Polarization in Dielectric and Magnetic Specimens of Limited Size Coming from LHI Materials

Below, these issues are investigated in the three formulations, ‘P-E, χ_e_’, ‘M-H, χ_m_’, and ‘P-D, χ_ε_’. In all cases, the important depolarizing effect is taken into account, which inevitably appears in all realistic dielectric and magnetic specimens due to their limited size [22,23,24,25,26,27,28,29,30,31,32]. Accordingly, *bound* electric and magnetic charges always appear at the surface of a realistic specimen (at least, if not even in the bulk) due to the discontinuity of the respective polarization, P(r,t) and $M\left(r,t\right)$. These *bound* charges behave as secondary sources producing the so-called *internal* field, whether we refer to electric [22,23,24,25,26] or magnetic [27,28,29,30,31,32] processes (the *internal* field is also called depolarizing/depolarization field or self-field). Surprisingly, different terminologies have been employed in the literature to distinguish these processes in electricity (depolarizing, depolarization, etc.) and magnetism (demagnetizing, demagnetization, etc.). Here, the single term ‘depolarizing’ is adopted to address the relevant processes/entities whether we refer to dielectric or magnetic materials since, after all, both P(r,t) and M(r,t) refer to the same physical entity, the polarization. Qualitatively, in both electricity and magnetism, the *internal*/depolarizing field opposes the external field, applied by the user, thus reducing its value, and tends to destroy (actually, to ‘self-destroy’) the polarization of the specimen. Quantitatively, in both electricity and magnetism, the produced *internal*/depolarizing field is expressed as a percentage of the parent vector field that is of the polarization, through the so-called depolarizing factor, N. The latter ranges within 0≤N≤1; apparently, the *internal* field can never exceed the parent field from which it is produced. Notably, the depolarizing factor, N, depends on a number of parameters: (i) the specific shape of the specimen, (ii) given the shape, the relative dimensions of the specimen, and (iii) the relative orientation between the specimen and the externally applied field [22,23,24,25,26,27,28,29,30,31,32].

A common basis of the discussion presented below in all three formulations, the ‘P-E, χ_e_’, ‘M-H, χ_m_’, and ‘P-D, χ_ε_’, is the description of the ‘transient stage’ during which the electric and magnetic polarization gradually develops after application of the relevant external field. To this effect, in all three cases, a schematic recursive sequence, termed ‘polarization cascade’, is employed. This scheme is reproduced mathematically in a detailed and consistent way by means of series. Though schematic, the ‘polarization cascade’, discussed below to describe the ‘transient stage’, is based on a solid physical basis. For instance, an analogous concept is the so-called response function, which, similarly, is used to describe the polarization of an LHI dielectric subjected to an external, time-dependent electric field [33]. This concept is further generalized to the Kronig–Kramers relations, which hold for many linear causal processes, including the polarization of dielectrics [15,26,33]. It should be noted that in the present Section 2, where a series-based approach is employed for the description of the transient stage, we restrict the discussion to the quasi-static limit of low frequency, ω. However, in Section 4, the fully time-dependent case is recovered without any restriction on the frequency, ω.

Before proceeding, to avoid any misunderstanding, we clarify that in the rest of the paper the following notation is adopted: in vector/scalar fields, upper indices refer to the investigated space in respect to the specimen (i.e., *in*: *interior* of the specimen, while *ex*: *exterior* of the specimen), while lower indices refer to the component of the vector/scalar field (i.e., *ext*: *external* applied by *free* charges, while *int*: *internal* produced by *bound* charges which relate to the polarization). When no lower index is used, the vector/scalar field is the total one. Finally, in the vector field of polarization, both electric, P, and magnetic, M, we do not use any upper/lower index because in all cases it obviously refers to the total physical entity, existing only at the interior of the specimen.

In Section 2.1, these issues are addressed for the ‘P-E, χ_e_’ formulation in LHI dielectric materials, while in Section 2.2, the ‘M-H, χ_m_’ formulation is used to explore the respective processes in LHI paramagnetic and diamagnetic materials. Finally, in Section 2.3, the strategy of the ‘M-H, χ_m_’ approach is adopted to introduce the relevant ‘P-D, χ_ε_’ formulation for the case of LHI dielectrics. It is shown that the ‘P-D, χ_ε_’ formulation restores the conceptual and series-associated flaws of the ‘P-E, χ_e_’ one. In addition, the absolute equivalence of the ‘P-D, χ_ε_’ and ‘P-E, χ_e_’ formulations is proved, while the complementary character of the respective depolarizing factors is shown through the generic condition $N_{e}+N_{\varepsilon }=1$.

### 2.1. Susceptibility and Polarization in Realistic Specimens of LHI Dielectric Materials: The ‘P-E, χ_e_’ Formulation

A series-based approach is employed here for the standard ‘P-E, χ_e_’ formulation to study the basic case of a dielectric specimen surrounded by vacuum. Accordingly, an *external* electric field, $E_{ext}(r,t)$, is applied to a realistic specimen originating from a parent LHI dielectric material. We aim to survey the endogenous properties, that is, the *intrinsic* susceptibility, $\chi_{e}$, of the parent LHI dielectric material. In the simple case, the *external* electric field is uniform, $E_{ext}\left(r,t\right)=E_{0}f(\omega t)\hat{z}$, where f(ωt) is a harmonic function of time, where the frequency, ω, is low so that in the below discussion, the quasi-static limit applies. The transient stage, when the response of the specimen to the *external* stimulus, $E_{ext}(r,t)$, still develops, is of particular interest. At these very first moments of the transient stage, $E_{ext}(r,t)$ penetrates the specimen, thus $E_{ext}^{in}\left(r,t\right)=E_{ext}(r,t)$, and initiates a ‘polarization cascade’; $E_{ext}(r,t)$ induces a first term in the polarization, $P\left(r,t\right)$, which produces a first term of the so-called *internal* electric field, $E_{int}^{in}\left(r,t\right)$. The latter induces a second term in the polarization, $P\left(r,t\right)$, which produces a second term of the internal electric field, $E_{int}^{in}\left(r,t\right)$, and so on. At the end of this recursive sequence, that is, at the steady state, both the polarization, $P\left(r,t\right)$, and the *internal* electric field, $E_{int}^{in}\left(r,t\right)$, are fixed. Then, $E_{int}^{in}\left(r,t\right)$ adds to $E_{ext}^{in}\left(r,t\right)=E_{ext}(r,t)$ to produce the total electric field $E^{in}\left(r,t\right)=E_{ext}^{in}\left(r,t\right)+E_{int}^{in}\left(r,t\right)$ at the interior of the dielectric specimen. Then, the following fundamental relation holds, $P\left(r,t\right)=\chi_{e}\varepsilon_{0}E^{in}\left(r,t\right)$. It should be recalled that the *internal* electric field, $E_{int}^{in}\left(r,t\right)$, is produced by the polarization *bound* charges, which in the general case are of volume, $\rho_{b}\left(r,t\right)=-\nabla \cdot P\left(r,t\right)$, and surface, $\sigma_{b}\left(r,t\right){|}_{S}=\hat{n}\cdot P\left(r,t\right){|}_{S}$, origin [8,9,10,11,12,13,14,15,16,17,18,19]. Below, we focus on the case where only surface charges exist (homogeneous $P\left(r,t\right)$) and utilize a series-based approach to find all $E^{in}\left(r,t\right)$, $E_{int}^{in}\left(r,t\right)$, $P\left(r,t\right),$ and $\sigma_{b}\left(r,t\right){|}_{S}$. Importantly, the depolarizing effect is taken into account, which inevitably appears in any dielectric specimen of limited size of a parent LHI material. The electric susceptibility of the parent LHI material is termed $\chi_{e}$ and is called the *intrinsic* electric susceptibility, $\chi_{e,intr}\equiv \chi_{e}$, since it stems from the endogenous physical properties of the LHI dielectric material per se. This is to be distinguished from the so-called *extrinsic* electric susceptibility, $\chi_{e,extr}$, that represents the exogenous properties of the particular specimen under investigation [22,23,24,25,26].

**Figure 1:**
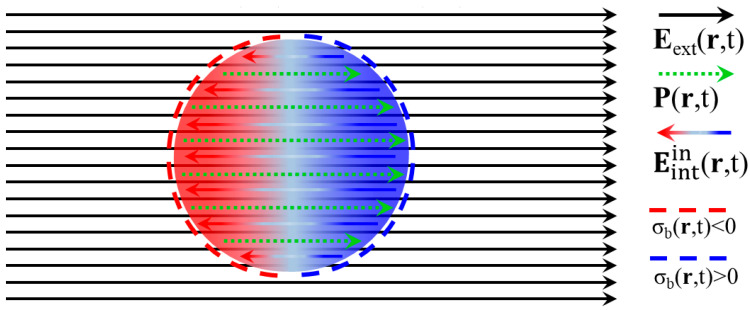
Schematic illustration of a simple spherical specimen of an LHI dielectric material, subjected to a uniform *external* electric field, $E_{ext}\left(r,t\right)=E_{0}f(\omega t)\hat{z}$, according to the ‘P-E, χ_e_’ formulation. The illustration focuses on the processes occurring at the interior of the specimen, so that the dipolar *internal* electric field at the exterior of the specimen, $E_{int}^{out}\left(r,t\right)$, is not shown. The color scale illustrates the spatial variation of the *internal* electric scalar potential, $U_{int}^{in}\left(r\right)=(N\chi_{e}/(1+N\chi_{e}))E_{0}z$, which produces $E_{int}^{in}\left(r,t\right)$ through $E_{int}^{in}\left(r,t\right)=-\nabla \cdot U_{int}^{in}\left(r,t\right)$ (see [21] and relation (34)).

The discussion of the present Section 2.1 will be based on the schematic illustration of Figure 1; the specimen is a dielectric sphere, well known for the homogeneous $P\left(r,t\right),$ which develops at its interior upon application of the uniform $E_{ext}\left(r,t\right)=E_{0}f(\omega t)\hat{z}$ (see below and [21]). Also, in this case, the depolarizing factor, N, can be calculated analytically. Obviously, the homogeneous polarization, $P\left(r,t\right)$, which is ultimately established at the interior of the specimen, is discontinuous at its surface; inevitably, only surface *bound* charges exist, having density $\sigma_{b}\left(r,t\right){|}_{S}=\hat{n}\cdot P\left(r,t\right){|}_{S}$ (see below and [21]). These produce the *internal* electric field, $E_{int}^{in}\left(r,t\right)$, which adds to the one, $E_{ext}^{in}\left(r,t\right)=E_{ext}\left(r,t\right)$, applied by the user so that the total electric field, $E^{n}\left(r,t\right)$, established at the interior of the specimen is $E^{in}\left(r,t\right)=E_{ext}^{in}\left(r,t\right)+E_{int}^{in}\left(r,t\right)$. Obviously, $E_{int}^{in}\left(r,t\right)$ is anti-parallel to both $P\left(r,t\right)$ and $E_{ext}^{in}\left(r,t\right)$. Finally, it is noted that the illustration focuses on the processes occurring at the interior of the specimen so that the respective dipolar *internal* electric field at the exterior of the specimen, $E_{int}^{out}\left(r,t\right)$, is not shown.

#### 2.1.1. Standard Calculations for the ‘P-E, χ_e_’ Formulation

We start by accessing the relevant vector fields of electricity by means of standard algebraic calculations. In the standard ‘P-E, χ_e_’ formulation, the *external* electric field, $E_{ext}(r,t)$, applied by the user controls the polarization of the specimen. Finally, that is at the permanent state, the polarization should be $P\left(r,t\right)=\chi_{e}\varepsilon_{0}E^{in}\left(r,t\right)$ where $E^{in}\left(r,t\right)$ is the total electric field at the interior of the specimen, that is

(29) $$E^{in}\left(r,t\right)=E_{ext}^{in}\left(r,t\right)+E_{int}^{in}\left(r,t\right)$$

where $E_{ext}^{in}\left(r\right)$ and $E_{int}^{in}\left(r\right)$ are the *external* component applied by the user and the *internal* component produced by the *bound* charges, of density $\sigma_{b}\left(r,t\right){|}_{S}$, which reside at the surface of the finite specimen due to the discontinuity of its polarization $P\left(r,t\right)$. It should be noted that the *internal* electric field, $E_{int}^{in}\left(r,t\right)$, opposes both the polarization, $P\left(r,t\right)$, and the *external* electric field, $E_{ext}^{in}\left(r,t\right)$. Since $E_{int}^{in}\left(r,t\right)$ is produced by the discontinuity of $P\left(r,t\right),$ a quantitative relation between these two vector fields can be employed. In our case of an LHI dielectric material, $E_{int}^{in}\left(r,t\right)$ and $P\left(r,t\right)$ relate through

(30) $$E_{int}^{in}\left(r,t\right)=-\left(\frac{N}{\varepsilon_{0}}\right)P\left(r,t\right)$$

where N is the so-called depolarizing factor, a scalar quantity [22,23,24,25,26]. By combining relations (9), (29), and (30), the following relation is easily obtained

(31) $$P\left(r,t\right)=\left(\frac{\chi_{e}}{1+N\chi_{e}}\right)\varepsilon_{0}E_{ext}^{in}\left(r,t\right)$$

The dimensionless proportionality factor of the two vector fields (i.e., excluding $\varepsilon_{0}$) is the so-called *extrinsic* electric susceptibility

(32) $$\chi_{e,extr}=\frac{\chi_{e}}{1+N\chi_{e}}$$

while $\chi_{e}$ is the *intrinsic* electric susceptibility ($\chi_{e}\equiv \chi_{e,intr}$), a quantity representative of the endogenous dielectric properties of the parent LHI material (not just of the specific specimen under investigation). The above relation can also be written in a form convenient to introduce an alternative definition of the depolarizing factor [22,23,24,25,26]

(33) $$N=\frac{1}{\chi_{e,extr}}-\frac{1}{\chi_{e}}$$

By combining the above relations (30) and (31), at the interior of the specimen, the *internal* electric field is obtained through the following relation

(34) $$E_{int}^{in}\left(r,t\right)=-\left(\frac{N\chi_{e}}{1+N\chi_{e}}\right)E_{ext}^{in}\left(r,t\right)$$

From the above relations, (30) and (34), it is concluded that, indeed, $E_{int}^{in}\left(r,t\right)$ opposes both $P\left(r,t\right)$ and $E_{ext}^{in}\left(r,t\right)$ (notice that 0≤N≤1 and $0\leq \chi_{e}<\infty$).

Finally, by using the above information, the total electric field $E^{in}\left(r,t\right)=E_{ext}^{in}\left(r,t\right)+E_{int}^{in}\left(r,t\right)$ is obtained from the following relation

(35) $$E^{in}\left(r,t\right)=\left(\frac{1}{1+N\chi_{e}}\right)E_{ext}^{in}\left(r,t\right)$$

Here, we realize that the factor $1/(1+N\chi_{e})$ which appears in the above relations (31), (32), (34), and (35), can be written as the sum of an infinite geometric series [34].

(36) $$\sum_{i=0}^{\infty }{\left(-{N\chi }_{e}\right)}^{i}=\frac{1}{1+N\chi_{e}}.$$

Under this substitution, relations (31), (32), (34), and (35) transform to the respective ones.

(37) $$P\left(r,t\right)=\left(\sum_{i=0}^{\infty }{\left(-{N\chi }_{e}\right)}^{i}\right)\chi_{e}\varepsilon_{0}E_{ext}^{in}\left(r,t\right)$$

(38) $$\chi_{e,extr}=\chi_{e}\left(\sum_{i=0}^{\infty }{\left(-{N\chi }_{e}\right)}^{i}\right)$$

(39) $$E_{int}^{in}\left(r,t\right)=-N\left(\sum_{i=0}^{\infty }{\left(-{N\chi }_{e}\right)}^{i}\right)\chi_{e}E_{ext}^{in}\left(r,t\right)$$

and

(40) $$E^{in}\left(r,t\right)=\left(\sum_{i=0}^{\infty }{\left(-{N\chi }_{e}\right)}^{i}\right)E_{ext}^{in}\left(r,t\right)$$

Formally speaking, the above relations are meaningful only when the geometric series of relation (36) converges, that is, only when the following condition holds [34].

(41) $$|-{N\chi }_{e}|<1$$

This fact is very crucial. It is discussed below in great detail in relation to the underlying physical processes of the transient stage, as realized by a schematic electric polarization cascade. Specifically, it is shown that the above quantitative, series-based, mathematical fact is reproduced on physical grounds by assuming that, upon application of the *external* field, the relevant polarization processes evolve through a recursive sequence during the transient stage, until the steady state is established. As shown below, this physical scheme addresses, in a detailed and accurate way, the mathematical counterpart presented above.

#### 2.1.2. Electric Field, $E\left(r,t\right)$, Based on a Scheme of Series

Following the scheme of series, the user-applied $E_{ext}(r,t)$ can be considered as the zeroth-order term, $E_{0}^{in}\left(r,t\right)$, of the total electric field, $E^{in}\left(r,t\right)$, which gradually develops during the initial stage (transient stage) and ultimately will be established at the interior of the dielectric specimen (steady state). The zeroth-order term, $E_{0}^{in}\left(r,t\right)$, will polarize, partially, the specimen by inducing a zeroth-order term of the electric polarization, $P_{0}\left(r,t\right)$, through $P_{0}\left(r,t\right)=\chi_{e}\varepsilon_{0}E_{0}^{in}\left(r,t\right)$. Generally, the polarization, $P\left(r,t\right)$, of a uniformly polarized dielectric specimen is accompanied by only a surface density of polarization *bound* charges, $\sigma_{b}\left(r,t\right){|}_{S}=\hat{n}\cdot P\left(r,t\right){|}_{S}$, which acts as a secondary source and produces an *internal* electric field $E_{int}^{in}\left(r,t\right)=-(N/\varepsilon_{0})P\left(r,t\right)$ at the interior of the dielectric specimen (please, recall that *N* is the depolarizing factor and ranges within 0≤N≤1 [22,23,24,25,26]). Accordingly, the zeroth-order term of the polarization $P_{0}\left(r,t\right)$ will produce a higher-order term, that is, the first-order term, in the *internal* electric field through $E_{int,1}^{in}\left(r,t\right)=-(N/\varepsilon )P_{0}\left(r,t\right)$. The first-order term, $E_{int,1}^{in}\left(r,t\right)$, will induce a same-order term, that is, the first-order term, in the polarization through $P_{1}\left(r,t\right)=\chi_{e}\varepsilon_{0}E_{int,1}^{in}\left(r,t\right)$. The latter will subsequently produce a second-order term in the *internal* electric field through $E_{int,2}^{in}\left(r,t\right)=-\left(N/\varepsilon_{0}\right)P_{1}\left(r,t\right),$ and so on. Generalizing this scheme, the (i-1)-order term of the induced polarization should be $P_{i-1}\left(r,t\right)=\chi_{e}\varepsilon_{0}E_{int,i-1}^{in}\left(r,t\right)$, whereas the (i)-order term of the *internal* electric field is given by $E_{int,i}^{in}\left(r,t\right)=-(N/\varepsilon_{0})P_{i-1}\left(r,t\right)$. The latter relations on $P_{i-1}\left(r,t\right)$ and $E_{int,i}^{in}\left(r,t\right)$ enables us to obtain the following relation.

(42) $$E_{int,i}^{in}\left(r,t\right)=\left(-{N\chi }_{e}\right)E_{int,i-1}^{in}\left(r,t\right)=\left(-{N\chi }_{e}\right)\left(-{N\chi }_{e}\right)E_{int,i-2}^{in}\left(r,t\right)=\;\left(-{N\chi }_{e}\right)\left(-{N\chi }_{e}\right)\left(-{N\chi }_{e}\right)E_{int,i-3}^{in}\left(r,t\right)=\ldots$$

else

(43) $$E_{int,i}^{in}\left(r,t\right)={\left(-{N\chi }_{e}\right)}^{i}E_{0}^{in}\left(r,t\right)$$

Now, it is easily seen that the total electric field, $E^{in}\left(r,t\right)$, at the interior of the dielectric specimen is described by an infinite series as follows:

(44) $$E^{in}\left(r,t\right)=E_{0}^{in}\left(r,t\right)+E_{int,1}^{in}\left(r,t\right)+E_{int,2}^{in}\left(r,t\right)+\ldots +E_{int,i}^{in}\left(r,t\right)+\ldots \;={\left(-{N\chi }_{e}\right)}^{0}E_{0}^{in}\left(r,t\right)+{\left(-{N\chi }_{e}\right)}^{1}E_{0}^{in}\left(r,t\right)+{\left(-{N\chi }_{e}\right)}^{2}E_{0}^{in}\left(r,t\right)+\ldots +{\left(-{N\chi }_{e}\right)}^{i}E_{0}^{in}\left(r,t\right)+\ldots$$

else

(45) $$E^{in}\left(r,t\right)=\left(\sum_{i=0}^{\infty }{\left(-{N\chi }_{e}\right)}^{i}\right)E_{0}^{in}\left(r,t\right)$$

where obviously $E_{0}^{in}\left(r,t\right)=E_{ext}\left(r,t\right)$.

This geometric series converges only when the following condition holds [34].

(46) $$|-N\chi_{e}|<1$$

resulting in

(47) $$\sum_{i=0}^{\infty }{\left(-{N\chi }_{e}\right)}^{i}=\frac{1}{1+N\chi_{e}}$$

Under these conditions, the total electric field at the interior of the specimen will be given by

(48) $$E^{in}\left(r,t\right)=\left(\frac{1}{1+N\chi_{e}}\right)E_{ext}\left(r,t\right).$$

Thus, recalling that $E_{ext}\left(r,t\right)=E_{ext}^{in}\left(r,t\right),$ it is easily seen that by using this scheme of series, relation (35) is recovered.

#### 2.1.3. Polarization, $P\left(r,t\right)$, Based on a Scheme of Series

The recursive sequence described above can be applied to the polarization, as well, as shown below. Accordingly, the polarization, $P\left(r,t\right)$, at the interior of the specimen, can be described by an infinite series. The zeroth-order term of $P\left(r,t\right)$ will be induced by the zeroth-order term of the electric field, $E_{0}^{in}\left(r,t\right)$, that is the *external* electric field, $E_{ext}\left(r,t\right)$, through $P_{0}\left(r,t\right)=\chi_{e}\varepsilon_{0}E_{0}^{in}\left(r,t\right)=\chi_{e}\varepsilon_{0}E_{ext}\left(r,t\right)$. It can easily be shown that, in general, the (i)-order term will be

(49) $$P_{i}\left(r,t\right)={\left(-{N\chi }_{e}\right)}^{i}\chi_{e}\varepsilon_{0}E_{ext}\left(r,t\right).$$

Accordingly, the polarization is given by

(50) $$P\left(r,t\right)=\sum_{i=0}^{\infty }P_{i}\left(r,t\right)=\left(\sum_{i=0}^{\infty }{\left(-{N\chi }_{e}\right)}^{i}\right)\chi_{e}\varepsilon_{0}E_{ext}\left(r,t\right).$$

As discussed above, this geometric series converges only when the condition of relation (46) is satisfied. Then, relation (50) obtains the form

(51) $$P\left(r,t\right)=\left(\frac{1}{1+N\chi_{e}}\right)\chi_{e}\varepsilon_{0}E_{ext}\left(r,t\right)=\left(\frac{\chi_{e}}{1+N\chi_{e}}\right)\varepsilon_{0}E_{ext}\left(r,t\right).$$

Recalling that $E_{ext}\left(r,t\right)=E_{ext}^{in}\left(r,t\right),$ it is seen that by using this scheme of series, relation (31) is recovered. Also, in this expression, the dimensionless proportionality factor of the two vector fields (i.e., excluding $\varepsilon_{0}$) is the *extrinsic* electric susceptibility $\chi_{e,extr}=\chi_{e}/\left(1+N\chi_{e}\right)$ already defined above, relation (32). Recall that $\chi_{e}$ is the *intrinsic* electric susceptibility ($\chi_{e}\equiv \chi_{e,intr}$), a quantity representative of the endogenous dielectric properties of the parent LHI material (not just of the specific specimen under investigation). Finally, above it was shown that $E^{in}\left(r,t\right)=E_{ext}(r,t)/(1+N\chi_{e})$, relation (48), so that relation (51) takes the equivalent form $P\left(r,t\right)=\chi_{e}\varepsilon_{0}E^{in}\left(r,t\right)$. Thus, through this series-based scheme, one recovers, in a consistent way, the relation that formally defines the polarization, $P\left(r,t\right)$, in respect to the total electric field, $E^{in}\left(r,t\right)$, at the interior of the specimen, according to the standard ‘P-E, χ_e_’ formulation discussed in this section.

#### 2.1.4. Bound Surface Charge Density, $\sigma_{b}\left(r,t\right){|}_{S}$, Based on a Scheme of Series for the ‘P-E, χ_e_’ Formulation

Finally, the same recursive sequence is applicable to the *bound* surface charge density, $\sigma_{b}\left(r,t\right){|}_{S}$, which gradually develops during the transient stage and eventually is established at the steady state at the surface of the dielectric specimen. The *bound* charge density, $\sigma_{b}\left(r,t\right){|}_{S}$, is induced by the primary source, the *free* charge density, and acts as a secondary source, thus producing the *internal* electric field, $E_{int}^{in}\left(r,t\right)$. The zeroth-order term of the polarization, $P_{0}\left(r,t\right)=\chi_{e}\varepsilon_{0}E_{0}^{in}\left(r,t\right)=\chi_{e}\varepsilon_{0}E_{ext}^{in}\left(r,t\right)$, induces a zeroth-order term in the *bound* surface charge density, $\sigma_{b,0}\left(r,t\right){|}_{S}=\hat{n}\cdot P_{0}\left(r,t\right){|}_{S}=\hat{n}\cdot (\chi_{e}\varepsilon_{0}E_{0}^{in}\left(r,t\right)){|}_{S}=\hat{n}\cdot \left(\chi_{e}\varepsilon_{0}E_{ext}^{in}\left(r,t\right)\right){|}_{S}$. It is expected that $\sigma_{b,0}\left(r,t\right){|}_{S}$ will produce a first-order term in the *internal* electric field, $E_{int,1}^{in}\left(r,t\right)$, inside the dielectric sphere. The latter is opposite to the *external* electric field, $E_{ext}(r,t)$. In accordance, $E_{int,1}^{in}\left(r,t\right)$ will induce a first-order term in the polarization, $P_{1}\left(r,t\right)$, which in turn will induce a first-order term in the *bound* surface charge density, $\sigma_{b,1}\left(r,t\right){|}_{S}=\hat{n}\cdot P_{1}\left(r,t\right){|}_{S}$. The latter produces the second-order term of the *internal* electric field, $E_{int,2}^{in}\left(r,t\right)$, and so on, until the steady state is established. It is easily seen that the (i)-order term of the *bound* surface charge density is given by $\sigma_{b,i}\left(r,t\right){|}_{S}=\hat{n}\cdot P_{i}\left(r,t\right){|}_{S}$, while the (i)-order term of the polarization is obtained through $P_{i}\left(r,t\right)={\left(-N\chi_{e}\right)}^{i}\chi_{e}\varepsilon_{0}E_{0}^{in}\left(r,t\right)$. As a consequence, the (i)-order term of the *bound* surface charge density is $\sigma_{b,i}\left(r,t\right){|}_{S}={\left(-N\chi_{e}\right)}^{i}\chi_{e}\varepsilon_{0}\hat{n}\cdot E_{0}^{in}\left(r,t\right){|}_{S}$. At the end, the complete *bound* surface charge density which builds up at the surface of the specimen is given by

(52) $$\sigma_{b}\left(r,t\right){|}_{S}=\sum_{i=0}^{\infty }\sigma_{b,i}\left(r,t\right){|}_{S}=\left(\sum_{i=0}^{\infty }{\left(-N\chi_{e}\right)}^{i}\right)\chi_{e}\varepsilon_{0}\hat{n}\cdot E_{0}^{in}\left(r,t\right){|}_{S}$$

else

(53) $$\sigma_{b}\left(r,t\right){|}_{S}=\left(\frac{1}{1+N\chi_{e}}\right)\varepsilon_{0}\chi_{e}\hat{n}\cdot E_{ext}\left(r,t\right){|}_{S}=\left(\frac{\chi_{e}}{1+N\chi_{e}}\right)\varepsilon_{0}\hat{n}\cdot E_{ext}\left(r,t\right){|}_{S}$$

Given that the convergence condition of relation (46) is satisfied. The exact same result can be obtained by other means. The *internal* electric field produced by $\sigma_{b}\left(r,t\right){|}_{S}$ is $E_{int}^{in}\left(r,t\right)=-(N\chi_{e}/(1+N\chi_{e}))E_{ext}(r,t)$. As expected, $E_{int}^{in}\left(r,t\right)$ opposes $E_{ext}\left(r,t\right)$ and relates to the polarization through $E_{int}^{in}\left(r,t\right)=-(N/\varepsilon_{0})P^{in}\left(r,t\right)$.

Summarizing the processes described for the standard ‘P-E, χ_e_’ formulation in the above Section 2.1.2, Section 2.1.3 and Section 2.1.4, the *external* electric field $E_{ext}\left(r,t\right)$ induces a polarization $P\left(r,t\right)=\left(1/\left(1+N\chi_{e}\right)\right)\chi_{e}\varepsilon_{0}E_{ext}\left(r,t\right)$ at the dielectric specimen. In turn, the discontinuity of $P\left(r,t\right)$ at the surface of the specimen establishes a *bound* surface charge density $\sigma_{b}\left(r,t\right){|}_{S}=(1/(1+N\chi_{e}))\varepsilon_{0}\chi_{e}\hat{n}\cdot E_{ext}(r,t){|}_{S},$ which produces an *internal* field $E_{int}^{in}\left(r,t\right)=-(N\chi_{e}/(1+N\chi_{e}))E_{ext}(r,t)$. The latter is inherently opposite to the *external* one, $E_{ext}\left(r,t\right)$. Accordingly, the *external* electric field, $E_{ext}\left(r,t\right)$, inside the dielectric specimen evolves to $E^{in}\left(r,t\right)=E_{ext}^{in}\left(r,t\right)+E_{int}^{in}\left(r,t\right)=(1/(1+N\chi_{e}))E_{ext}(r,t)$. Since the *internal* electric field, $E_{int}^{in}\left(r,t\right)$, is opposite to the *external* one, at the interior of the specimen, the relation $E^{in}\left(r,t\right)<E_{ext}\left(r,t\right)$, always holds. In addition, obviously $E_{int}^{in}\left(r,t\right)$ clearly acts toward the depolarization of the dielectric specimen [22,23,24,25,26]. This is why it is commonly termed as a depolarizing field or self field (see [8] pages 93–96; [14] pages 127, 158 and § 6.6.2; [15] page 24). Finally, it should be underlined that these processes appear due to the finite size of the specimen. In the ideal case of an infinite specimen, the lack of external surfaces will result in $\sigma_{b}\left(r,t\right){|}_{S}=0$ and $E_{int}^{in}\left(r,t\right)=0$, so that the electric field at the interior of the specimen will simply be equal to the *externally* applied one, $E^{in}\left(r,t\right)=E_{ext}\left(r,t\right)$. In the more realistic case of a ‘large’ specimen, the surfaces are placed far enough so that the $E_{int}^{in}\left(r,t\right)$ produced by the respective non-zero $\sigma_{b}\left(r,t\right){|}_{S}$ is negligibly small in most parts of the specimen. Equivalently, the same conclusions are reached when the depolarizing factor is taken to be zero, N=0.

#### 2.1.5. Non-Compliance of the ‘P-E, χ_e_’ Formulation with the Convergence Condition of the Series

The series-based scheme employed above to describe the evolution of polarization under application of an external stimulus restores, somehow, the conceptually misleading causality between $P\left(r,t\right)$ and $E\left(r,t\right)$ (this conceptual flaw is inherent in the standard ‘P-E, χ_e_’ formulation as discussed, e.g., in [8] on pages 68 and 76 and in [13] on page 186). Yet, a serious algebraic hurdle still remains since in strict mathematical terms the geometric series met in all cases above should converge only when $|-N\chi_{e}|<1$, as already discussed above. By recalling that 0≤N≤1 and $0\leq \chi_{e}<\infty$, the above condition implies that when the depolarizing factor, N, is fixed (i.e., the shape and dimensions of the specific specimen under investigation), the electric susceptibility, $\chi_{e}$, should be restricted within $0\leq \chi_{e}<1/N$. On the other hand, in the standard ‘P-E, χ_e_’ formulation employed today, $0\leq \chi_{e}<\infty$ by definition. Curiously, this contradictory point of the ‘P-E, χ_e_’ formulation is not even mentioned in the literature, no objections are raised on the applicability of the obtained solutions, and we keep using this rather ill-defined electric susceptibility, χ_e_, in the whole range $0\leq \chi_{e}<\infty$.

Typical values of the relative permittivity for representative dielectric substances are presented in Appendix A, together with the basics of electric polarization [35,36,37,38,39,40,41]. From these data, it is seen that substances in the gas phase exhibit values of relative permittivity, $\varepsilon_{r}<2$, so that the respective susceptibility, $\chi_{e}=\varepsilon_{r}-1<1$, satisfies the condition $|-N\chi_{e}|<1$ in the entire range 0≤N≤1. However, practically, almost all liquids and solids exhibit relative permittivity, $\varepsilon_{r}>2$, so that the respective susceptibility, $\chi_{e}=\varepsilon_{r}-1>1$. Thus, the convergence condition $|-N\chi_{e}|<1$ is not satisfied by default in the entire range 0≤N≤1; it is satisfied only within $0\leq N\leq 1/\chi_{e}$. As a consequence, the ‘P-E, χ_e_’ formulation does not conform entirely with the convergence condition $|-N\chi_{e}|<1$.

### 2.2. Susceptibility and Polarization in Realistic Specimens of LHI Magnetic Materials: The ‘M-H, χ_m_’ Formulation

Our discussion now continues with magnetism, by considering the case of a realistic specimen with limited size coming from a parent LHI magnetic material. The respective series-based approach is employed here for the standard ‘M-H, χ_m_’ formulation to study the basic case of a magnetic specimen surrounded by vacuum when subjected to an *external* magnetic field, $H_{ext}(r,t)$. Again, the endogenous properties, that is, the *intrinsic* magnetic susceptibility, $\chi_{m}$, of the parent LHI magnetic material is surveyed. Also, the basic case of an *external* uniform, time-harmonic magnetic field, $H_{ext}\left(r,t\right)=H_{0}f(\omega t)\hat{z}$, is considered, where the frequency, ω, is low so that the quasi-static limit applies. At the transient stage, $H_{ext}(r,t)$ penetrates the specimen and initiates the ‘polarization cascade’; $H_{ext}(r,t)$ induces a first term in the polarization, $M\left(r,t\right)$, which produces a first term of the so-called *internal* magnetic field, $H_{int}^{in}\left(r,t\right)$, in turn inducing a second term in the polarization, which produces a second term of the internal electric field, and so on. At the end of this recursive sequence where the steady state is established, both the polarization, $M\left(r,t\right)$, and the *internal* magnetic field, $H_{int}^{in}\left(r,t\right)$, are fixed at the interior of the specimen. Then, $H_{int}^{in}\left(r,t\right)$ adds to $H_{ext}^{in}\left(r,t\right)=H_{ext}(r,t)$ to produce the total magnetic field $H^{in}\left(r,t\right)=H_{ext}^{in}\left(r,t\right)+H_{int}^{in}\left(r,t\right)$ at the interior of the magnetic specimen, so that the standard relation holds, $M\left(r,t\right)=\chi_{m}H^{in}\left(r,t\right)$. It should be recalled that the *internal* magnetic field, $H_{int}^{in}\left(r,t\right)$ is produced by the polarization *bound* (pseudo) charges, which in the general case are of volume, $\rho_{b,m}\left(r,t\right)=-\nabla \cdot M\left(r,t\right)$, and surface, $\sigma_{b,m}\left(r,t\right){|}_{S}=\hat{n}\cdot M\left(r,t\right){|}_{S}$, origin [8,9,10,11,12,13,14,16,17,18,19,31,32]. As in the case of the ‘P-E, χ_e_’ formulation above, we focus only on surface (pseudo) charges and utilize the series-based approach to find all $H^{in}\left(r,t\right)$, $H_{int}^{in}\left(r,t\right)$, $M\left(r,t\right),$ and $\sigma_{b,m}\left(r,t\right){|}_{S}$. Also, the depolarizing effect is taken into account, which apparently appears in any magnetic specimen of limited size. The magnetic susceptibility of the parent LHI material is simply termed $\chi_{m}$ and is called the *intrinsic* magnetic susceptibility, $\chi_{m,intr}\equiv \chi_{m}$, since it stems from the endogenous physical properties of the material per se. This is to distinguish from the so-called *extrinsic* magnetic susceptibility, $\chi_{m,extr}$, of the respective specimen under investigation, which apparently has limited size (see below).

**Figure 2:**
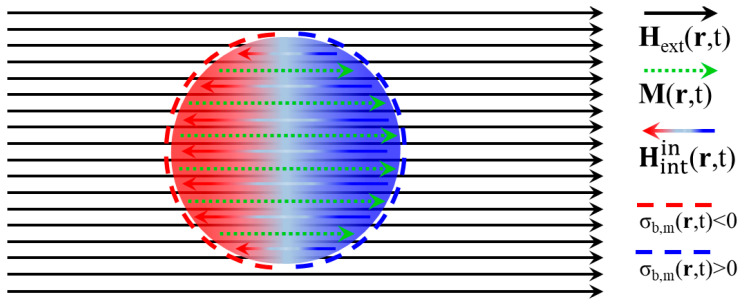
Schematic illustration of a simple spherical specimen of an LHI magnetic material, subjected to a uniform *external* magnetic field, $H_{ext}\left(r,t\right)=H_{0}f(\omega t)\hat{z}$, according to the ‘M-H, χ_m_’ formulation. Though in general, $-1\leq \chi_{m}<\infty$, here, for brevity, only the paramagnetic case is presented where $0\leq \chi_{m}$ (the diamagnetic case, $-1\leq \chi_{m}\leq 0$, can be illustrated analogously). Also, the illustration focuses on the processes occurring at the interior of the specimen so that the dipolar *internal* magnetic field at the exterior of the specimen, $H_{int}^{out}\left(r,t\right)$, is not shown. The color scale illustrates the spatial variation of the *internal* magnetic scalar (pseudo) potential, $U_{m,int}^{in}\left(r\right)=(N\chi_{m}/(1+N\chi_{m}))H_{0}z$, which produces $H_{int}^{in}\left(r,t\right)$ through $H_{int}^{in}\left(r,t\right)=-\nabla \cdot U_{m,int}^{in}\left(r,t\right)$ (see relation (59)).

The discussion of the present Section 2.2 holds for both cases of negative magnetic susceptibility, $-1\leq \chi_{m}\leq 0$ (diamagnetic LHI substances) and positive magnetic susceptibility, $0\leq \chi_{m}$ (paramagnetic and probably some soft ferromagnetic LHI substances). The schematic illustration of Figure 2 refers only to the particular specimen of a paramagnetic sphere, so that $0\leq \chi_{m}$ (the case of a diamagnetic sphere with $-1\leq \chi_{m}\leq 0$ can be illustrated analogously). The sphere exhibits homogeneous $M\left(r,t\right)$ which develops at its interior upon application of the uniform $H_{ext}\left(r,t\right)=H_{0}f(\omega t)\hat{z}$ (see below). Also, in the case of the sphere presented here, the depolarizing factor, N, can be calculated analytically. Obviously, the homogeneous polarization, $M\left(r,t\right)$, which ultimately establishes that the interior of the specimen is discontinuous at its surface so that only surface *bound* (pseudo) charges exist with density $\sigma_{b,m}\left(r,t\right){|}_{S}=\hat{n}\cdot M\left(r,t\right){|}_{S}$. These produce the *internal* magnetic field, $H_{int}^{in}\left(r,t\right)$, which adds to the *external* one, $H_{ext}^{in}\left(r,t\right)=H_{ext}\left(r,t\right)$, applied by the user so that the total magnetic field, $H^{in}\left(r,t\right)$, at the interior of the specimen is $H^{in}\left(r,t\right)=H_{ext}^{in}\left(r,t\right)+H_{int}^{in}\left(r,t\right)$. Obviously, $H_{int}^{in}\left(r,t\right)$ is anti-parallel to both $M\left(r,t\right)$ and $H_{ext}^{in}\left(r,t\right)$. Finally, it is noted that the illustration focuses on the processes occurring at the interior of the paramagnetic specimen so that the respective dipolar *internal* magnetic field at the exterior of the specimen, $H_{int}^{out}\left(r,t\right)$, is not shown.

#### 2.2.1. Standard Calculations for the ‘M-H, χ_m_’ Formulation

Our discussion starts by accessing the relevant vector fields by standard algebraic calculations. The *external* magnetic field, $H_{ext}(r,t)$, applied by the user is the cause that initiates the polarization of the specimen, which finally, at the permanent state, becomes

(54) $$M\left(r,t\right)=\chi_{m}H^{in}\left(r,t\right)$$

with $H^{in}\left(r,t\right)$ the total magnetic field at the interior of the specimen, that is

(55) $$H^{in}\left(r,t\right)=H_{ext}^{in}\left(r,t\right)+H_{int}^{in}\left(r,t\right)$$

where $H_{ext}^{in}\left(r\right)$ and $H_{int}^{in}\left(r\right)$ are the two components of the magnetic field existing at the interior of the specimen. The first is the *external* one applied by the user, while the second is the *internal* one produced by the magnetic (pseudo) charges, of density $\sigma_{b,m}\left(r,t\right){|}_{S}$, which reside at the surface of the finite specimen due to the discontinuity of its polarization, $M\left(r,t\right)$. It should be noted that the *internal* magnetic field, $H_{int}^{in}\left(r,t\right)$, opposes both the polarization, $M\left(r,t\right)$, and the *external* magnetic field, $H_{ext}^{in}\left(r,t\right)$. Once $H_{int}^{in}\left(r,t\right)$ is produced by the discontinuity of $M\left(r,t\right),$ a quantitative relation between these two vector fields can be employed. In our case, an LHI magnetic material, $H_{int}^{in}\left(r,t\right)$ and $M\left(r,t\right)$ relate through

(56) $$H_{int}^{in}\left(r,t\right)=-NM\left(r,t\right)$$

where N is the so-called depolarizing factor, a scalar quantity [27,28,29,30,31,32]. By combining the above relations (54)–(56), the following relation is easily obtained

(57) $$M\left(r,t\right)=\left(\frac{\chi_{m}}{1+N\chi_{m}}\right)H_{ext}^{in}\left(r,t\right)$$

The dimensionless proportionality factor of the two vector fields is the so-called *extrinsic* magnetic susceptibility

(58) $$\chi_{m,extr}=\frac{\chi_{m}}{1+N\chi_{m}}$$

while we recall that $\chi_{m}$ is the *intrinsic* magnetic susceptibility ($\chi_{m}\equiv \chi_{m,intr}$), a quantity representative of the endogenous magnetic properties of the parent LHI material (not just of the specific specimen under investigation).

By combining the above relations (56) and (57), at the interior of the specimen, the *internal* magnetic field can be obtained through the relation.

(59) $$H_{int}^{in}\left(r,t\right)=-\left(\frac{N\chi_{m}}{1+N\chi_{m}}\right)H_{ext}^{in}\left(r,t\right)$$

From the above relations (56) and (59), it is concluded that, indeed, $H_{int}^{in}\left(r,t\right)$ opposes both $M\left(r,t\right)$ the $H_{ext}^{in}\left(r,t\right)$ (notice that 0≤N≤1 and for the paramagnetic case considered here $0\leq \chi_{m}$).

Finally, by using the above relations (55) and (59), it is easily seen that at the interior of the specimen, the total magnetic field, $H^{in}\left(r,t\right)$, follows the relation

(60) $$H^{in}\left(r,t\right)=\left(\frac{1}{1+N\chi_{m}}\right)H_{ext}^{in}\left(r,t\right)$$

As was discussed in Section 2.1 for the ‘P-E, χ_e_’ formulation, here, for the case of the ‘M-H, χ_m_’ one, the factor $1/(1+N\chi_{m})$ can be written in an analogous fashion as the sum of an infinite geometric series [34].

(61) $$\sum_{i=0}^{\infty }{\left(-{N\chi }_{m}\right)}^{i}=\frac{1}{1+N\chi_{m}}$$

Under this substitution, relations (57)–(60) transform to the respective ones.

(62) $$M\left(r,t\right)=\left(\sum_{i=0}^{\infty }{\left(-{N\chi }_{m}\right)}^{i}\right)\chi_{m}H_{ext}^{in}\left(r,t\right)$$

(63) $$\chi_{m,extr}=\chi_{m}\left(\sum_{i=0}^{\infty }{\left(-{N\chi }_{m}\right)}^{i}\right)$$

(64) $$H_{int}^{in}\left(r,t\right)=-N\left(\sum_{i=0}^{\infty }{\left(-{N\chi }_{m}\right)}^{i}\right)\chi_{m}H_{ext}^{in}\left(r,t\right)$$

and

(65) $$H^{in}\left(r,t\right)=\left(\sum_{i=0}^{\infty }{\left(-{N\chi }_{m}\right)}^{i}\right)H_{ext}^{in}\left(r,t\right)$$

From a purely algebraic viewpoint, the above relations are meaningful only when the geometric series converges, that is, only when the following condition holds [34].

(66) $$|-N\chi_{m}|<1$$

As in Section 2.2 above for the case of the ‘P-E, χ_e_’ formulation, below we discuss for the ‘M-H, χ_m_’ one, the underlying physical processes of the transient stage, realized by a schematic magnetic polarization cascade.

#### 2.2.2. Magnetic Field, $H\left(r,t\right)$, Based on a Scheme of Series

Our discussion starts with the total magnetic field, $H^{in}\left(r,t\right)$, at the interior of the specimen. Based on the series scheme, the *external* magnetic field, $H_{ext}(r,t)$, applied by the user, can be considered as the zeroth-order term, $H_{0}^{in}\left(r,t\right)$, of the total magnetic field, $H^{in}\left(r,t\right)=H_{ext}^{in}\left(r,t\right)+H_{int}^{in}\left(r,t\right)$, that gradually develops (transient state) and eventually will be established (steady state) at the interior of the magnetic specimen. Accordingly, the zeroth-order term, $H_{0}^{in}\left(r,t\right)$, will polarize, partially, the specimen. The respective zeroth-order term of the polarization, $M_{0}^{in}\left(r,t\right)$, induced by $H_{0}^{in}\left(r,t\right)$, is $M_{0}^{in}\left(r,t\right)=\chi_{m}H_{0}^{in}\left(r,t\right)$.

In the general case, the polarization, $M\left(r,t\right)$, of a uniformly polarized specimen relates to a surface density of polarization *bound* magnetic (pseudo) charges, $\sigma_{b,m}\left(r,t\right){|}_{S}=\hat{n}\cdot M\left(r,t\right){|}_{S}$. The latter acts as a secondary source producing an *internal* magnetic field (depolarizing field) $H_{int}^{in}\left(r,t\right)=-NM\left(r,t\right)$ at the interior of the specimen (relation (56), above).

Accordingly, the zeroth-order term of the polarization $M_{0}\left(r,t\right)$ will produce a first-order term for the *internal* magnetic field given by $H_{int,1}^{in}\left(r,t\right)=-NM_{0}\left(r,t\right)$ (notice that the term $H_{int,0}^{in}\left(r,t\right)$ does not exist; the only zeroth-order magnetic field term is of *external* origin, $H_{0}^{in}\left(r,t\right)$). In turn, the first-order term, $H_{int,1}^{in}\left(r,t\right)$, will induce a first-order term in the polarization $M_{1}\left(r,t\right)=\chi_{m}H_{int,1}^{in}\left(r,t\right),$ which subsequently will produce a second-order term for the *internal* magnetic field $H_{int,2}^{in}\left(r,t\right)=-NM_{1}\left(r,t\right),$ and so on. In general, the (i-1)-order term of the induced polarization is $M_{i-1}\left(r,t\right)=\chi_{m}H_{int,i-1}^{in}\left(r,t\right)$, while the (i)-order term of the *internal* magnetic field is $H_{int,i}^{in}\left(r,t\right)=-NM_{i-1}\left(r,t\right)$. Combining these last two relations of $M_{i-1}\left(r,t\right)$ and $H_{int,i}^{in}\left(r,t\right),$ the following relation is obtained.

(67) $$H_{int,i}^{in}\left(r,t\right)=\left(-{N\chi }_{m}\right)H_{int,i-1}^{in}\left(r,t\right)=\left(-{N\chi }_{m}\right)\left(-{N\chi }_{m}\right)H_{int,i-2}^{in}\left(r,t\right)=\;\left(-{N\chi }_{m}\right)\left(-{N\chi }_{m}\right)\left(-{N\chi }_{m}\right)H_{int,i-3}^{in}\left(r,t\right)=\ldots$$

else

(68) $$H_{int,i}^{in}\left(r,t\right)={\left(-{N\chi }_{m}\right)}^{i}H_{0}^{in}\left(r,t\right)$$

Thus, the total magnetic field at the interior of the specimen, $H^{in}\left(r,t\right)$, will be given by the following infinite series

(69) $$H^{in}\left(r,t\right)=H_{0}^{in}\left(r,t\right)+H_{int,1}^{in}\left(r,t\right)+H_{int,2}^{in}\left(r,t\right)+\ldots +H_{int,i}^{in}\left(r,t\right)+\ldots \;={\left(-{N\chi }_{m}\right)}^{0}H_{0}^{in}\left(r,t\right)+{\left(-{N\chi }_{m}\right)}^{1}H_{0}^{in}\left(r,t\right)+{\left(-{N\chi }_{m}\right)}^{2}H_{0}^{in}\left(r,t\right)+\ldots \;+{\left(-{N\chi }_{m}\right)}^{i}H_{0}^{in}\left(r,t\right)+\ldots$$

else

(70) $$H^{in}\left(r,t\right)=\left(\sum_{i=0}^{\infty }{\left(-{N\chi }_{m}\right)}^{i}\right)H_{ext}\left(r,t\right)$$

where it is recalled that $H_{0}^{in}\left(r,t\right)=H_{ext}^{in}\left(r,t\right)$. Also, recalling that $H_{ext}^{in}\left(r,t\right)=H_{ext}\left(r,t\right),$ it can be easily shown that this relation is identical to relation (65) obtained above.

The above geometric series results in

(71) $$\sum_{i=0}^{\infty }{\left(-{N\chi }_{m}\right)}^{i}=\frac{1}{1+N\chi_{m}}$$

As already discussed, this result should formally hold when the convergence condition $|-N\chi_{m}|<1$, relation (66), is satisfied [34]. Then, the total magnetic field at the interior of the specimen will be

(72) $$H^{in}\left(r,t\right)=\left(\frac{1}{1+N\chi_{m}}\right)H_{ext}\left(r,t\right).$$

Recalling that $H_{ext}\left(r,t\right)=H_{ext}^{in}\left(r,t\right),$ it is easily seen that this relation is identical to relation (60) obtained above.

#### 2.2.3. Polarization, **M**(**r**,t), Based on a Scheme of Series

The series scheme discussed above applies, also, to the polarization. Indeed, here it is shown that the polarization, at the interior of the specimen, $M\left(r,t\right)$, results from an infinite series in the same recursive process described above. Thus, the zeroth-order term of the polarization will be $M_{0}\left(r,t\right)=\chi_{m}H_{0}^{in}\left(r,t\right)=\chi_{m}H_{ext}\left(r,t\right)$. It is easily shown that, in general, the (i)-order term will be

(73) $$M_{i}\left(r,t\right)={\left(-{N\chi }_{m}\right)}^{i}\chi_{m}H_{ext}\left(r,t\right).$$

Accordingly, the polarization is given by

(74) $$M\left(r,t\right)=\sum_{i=0}^{\infty }M_{i}\left(r,t\right)=\left(\sum_{i=0}^{\infty }{\left(-{N\chi }_{m}\right)}^{i}\right)\chi_{m}H_{ext}\left(r,t\right).$$

This relation is identical to relation (62) obtained above. Strictly speaking, only when the convergence condition $|-N\chi_{m}|<1$ holds [34], the above relation (74) takes the familiar form

(75) $$M\left(r,t\right)=\left(\frac{\chi_{m}}{1+N\chi_{m}}\right)H_{ext}\left(r,t\right)$$

which is identical to relation (57) obtained above, since $H_{ext}\left(r,t\right)=H_{ext}^{in}\left(r,t\right)$.

Note that, once it was shown that $H^{in}\left(r,t\right)=H_{ext}(r,t)/(1+N\chi_{m})$, relation (72), the above relation (75) takes the equivalent, well-known form

(76) $$M\left(r,t\right)=\chi_{m}H^{in}\left(r,t\right)$$

where $\chi_{m}$ is the *intrinsic* magnetic susceptibility which represents the endogenous properties of the parent LHI material. Indeed, this is the relation that formally defines the polarization, $M\left(r,t\right)$, in respect to the total magnetic field, $H^{in}\left(r,t\right)$, at the interior of the specimen, according to the standard M-H, $\chi_{m}$ formulation employed today [8,9,10,11,12,13,14,15].

Finally, given that $\chi_{m,extr}=\chi_{m}/(1+N\chi_{m})$, relation (58), the above relation (75) transforms to

(77) $$M\left(r,t\right)=\chi_{m,extr}H_{ext}\left(r,t\right)$$

where we recall that the dimensionless proportionality factor is the so-called *extrinsic* magnetic susceptibility which represents the exogenous properties of the particular specimen under investigation. Indeed, this is a conceptually reasonable definition of $\chi_{m,extr}$; a scalar constant which relates the polarization, $M\left(r,t\right)$, of the magnetic specimen to the *external* magnetic field, $H_{ext}\left(r,t\right)$ (not to the total magnetic field, $H\left(r,t\right)$).

#### 2.2.4. Bound Surface (Pseudo) Charge Density, $\sigma_{b,m}\left(r,t\right){|}_{S}$, Based on a Scheme of Series for the ‘M-H, χ_m_’ Formulation

Finally, the series scheme also applies to the surface density of *bound* magnetic pseudocharges, $\sigma_{b,m}\left(r,t\right){|}_{S}$, which gradually develops (transient state) and eventually will be established (steady state) at the surface, S, of the magnetic specimen (due to the discontinuity of its polarization, $M\left(r,t\right)$). As already discussed above, $\sigma_{b,m}\left(r,t\right){|}_{S}$ acts as a secondary source and ultimately produces inside the specimen the *internal* magnetic field, $H_{int}^{in}\left(r,t\right)$. Specifically, the zeroth-order term of the polarization, $M_{0}\left(r,t\right)=\chi_{m}H_{0}^{in}\left(r,t\right)=\chi_{m}H_{ext}^{in}\left(r,t\right)$, will induce a zeroth-order term $\sigma_{b,m,0}\left(r,t\right){|}_{S}=\hat{n}\cdot M_{0}\left(r,t\right){|}_{S}=\hat{n}\cdot (\chi_{m}H_{0}^{in}\left(r,t\right)){|}_{S}=\hat{n}\cdot \left(\chi_{m}H_{ext}^{in}\left(r,t\right)\right){|}_{S}$. Then, $\sigma_{b,m,0}\left(r,t\right){|}_{S}$ will produce a first-order term of the *internal* magnetic field, $H_{int,1}^{in}\left(r,t\right)$, at the interior of the specimen. Also, $H_{int,1}^{in}\left(r,t\right)$ will induce a first-order term in the polarization, $M_{1}^{in}\left(r,t\right)$, which in turn induces a first-order term in the surface density of *bound* magnetic (pseudo) charges, $\sigma_{b,m,1}\left(r,t\right){|}_{S}=\hat{n}\cdot M_{1}\left(r,t\right){|}_{S}$, responsible for the second-order term of the *internal* magnetic field, $H_{int,2}^{in}\left(r,t\right)$, and so on. It can be easily shown that the (i)-order term of the surface density of *bound* magnetic pseudocharges is $\sigma_{b,m,i}\left(r,t\right){|}_{S}=\hat{n}\cdot M_{i}^{in}\left(r,t\right){|}_{S}$. As shown above, the (i)-order term of the polarization is $M_{i}\left(r,t\right)={\left(-N\chi_{m}\right)}^{i}\chi_{m}H_{0}^{in}\left(r,t\right)$. Thus, the (i)-order term of the surface density of *bound* magnetic (pseudo) charges is $\sigma_{b,m,i}\left(r,t\right){|}_{S}={\left(-N\chi_{m}\right)}^{i}\chi_{m}\hat{n}\cdot H_{0}^{in}\left(r,t\right){|}_{S}$. Eventually, the complete surface density of *bound* magnetic (pseudo) charges established at the specimen surface is

(78) $$\sigma_{b,m}\left(r,t\right){|}_{S}=\sum_{i=0}^{\infty }\sigma_{b,m,i}\left(r,t\right){|}_{S}=\left(\sum_{i=0}^{\infty }{\left(-N\chi_{m}\right)}^{i}\right)\chi_{m}\hat{n}\cdot H_{0}^{in}\left(r,t\right){|}_{S}$$

else

(79) $$\sigma_{b,m}\left(r,t\right){|}_{S}=\left(\frac{1}{1+N\chi_{m}}\right)\chi_{m}\hat{n}\cdot H_{ext}\left(r,t\right){|}_{S}=\left(\frac{\chi_{m}}{1+N\chi_{m}}\right)\hat{n}\cdot H_{ext}\left(r,t\right){|}_{S}$$

which, as usual, holds when the convergence condition $|-N\chi_{m}|<1$ is satisfied [34]. This is the exact result that can be obtained by standard calculations. The *internal* magnetic field, ultimately produced by $\sigma_{b,m}\left(r,t\right){|}_{S}$, is $H_{int}^{in}\left(r,t\right)=-(N\chi_{m}/(1+N\chi_{m}))H_{ext}(r,t)$ and relates to the polarization through $H_{int}^{in}\left(r,t\right)=-NM\left(r,t\right)$. Summarizing the processes described above for the total magnetic field, $H^{in}\left(r,t\right)$, magnetic polarization, $M\left(r,t\right)$ and surface density of *bound* magnetic (pseudo) charges, $\sigma_{b,m}\left(r,t\right){|}_{S}$, the application of the *external* magnetic field $H_{ext}\left(r,t\right)$, in the steady state, establishes at the interior of the specimen, a polarization $M\left(r,t\right)=\chi_{m}\left(1/\left(1+N\chi_{m}\right)\right)H_{ext}\left(r,t\right)$ which induces a surface density of *bound* magnetic (pseudo) charges, $\sigma_{b,m}\left(r,t\right){|}_{S}=(\chi_{m}/(1+N\chi_{m}))\hat{n}\cdot H_{ext}(r,t){|}_{S}$. In turn, the latter produces an *internal* magnetic field, $H_{int}^{in}\left(r,t\right)=-(N\chi_{m}/(1+N\chi_{m}))H_{ext}(r,t)$, inherently opposite to the *external* one, $H_{ext}\left(r,t\right)$. Thus, at the interior of the specimen, the *external* magnetic field, $H_{ext}\left(r,t\right)$, evolves to $H^{in}\left(r,t\right)=(1/(1+N\chi_{m}))H_{ext}(r,t)$. Obviously, the *internal* magnetic field, $H_{int}^{in}\left(r,t\right)$, always opposes the *external* one so that, at the interior of the specimen, the total magnetic field is always lower than the *external* one applied by the user, $H^{in}\left(r,t\right)<H_{ext}\left(r,t\right)$. In addition, it is evident that $H_{int}^{in}\left(r,t\right)$ actually acts toward the depolarization of the magnetic specimen [27,28,29,30,31,32]. Finally, it should be stressed that these processes take place due to the finite size of the specimen. Ideally, in an infinite specimen, the lack of external surfaces will result in $\sigma_{b,m}\left(r,t\right){|}_{S}=0$ and $H_{int}^{in}\left(r,t\right)=0$, so that the magnetic field inside the specimen will be equal to the *externally* applied one, $H^{in}\left(r\right)=H_{ext}\left(r\right)$. More realistically, in a ‘large’ specimen, the surfaces are placed far enough so that the $H_{int}^{in}\left(r,t\right)$ produced by the respective non-zero $\sigma_{b,m}\left(r,t\right){|}_{S}$ is negligibly small in most parts of the specimen. Equivalently, the same conclusions are reached when the depolarizing factor is taken to be zero, N=0.

#### 2.2.5. Compliance of the ‘M-H, χ_m_’ Formulation with the Convergence Condition of the Series

The mathematical simulation of the physical recursive sequence, by using the series scheme discussed above in detail, is revealing. Notably, the factor $\left(1/(1+N\chi_{m})\right)$ appears in the expressions of all $H^{in}\left(r,t\right)$, $H_{int}^{in}\left(r,t\right)$, $M\left(r,t\right),$ and $\sigma_{b,m}\left(r,t\right){|}_{S}$. An interesting consequence is that from a purely algebraic point of view, these physical entities should be formally definable only when the following convergence condition $|-N\chi_{m}|<1$, relation (66), is satisfied.

Here, the possible implications that this fact may have on the physics of the underlying magnetic polarization processes are discussed. First, recall that N is the depolarizing factor, a technical coefficient of *extrinsic* origin which depends on a number of parameters of the specific specimen under investigation: (i) its shape, (ii) its relative dimensions, and (iii) its relative orientation with respect to the *external* field [27,28,29,30,31,32]. However, N does not depend on the endogenous physical properties of the parent LHI magnetic material. Second, as already discussed above, $\chi_{m}$ is the magnetic susceptibility, a physical parameter of *intrinsic* origin that depends on the endogenous properties of the parent LHI magnetic material (but not on the demagnetizing factor of the specific specimen). Accordingly, these two independent parameters, N and $\chi_{m}$, should be engaged so that the convergence condition $|-N\chi_{m}|<1$, relation (66), is satisfied. In this context, recall that N ranges within 0≤N≤1 [27,28,29,30,31,32]. Furthermore, in the most general case (i.e., when all magnetic materials, even non-linear ones, are included, starting from diamagnets up to hard ferromagnets), $\chi_{m}$ is defined within $-1\leq \chi_{m}<\infty$. However, here, only LHI materials are addressed, so our discussion should formally be restricted to paramagnetic ones with $0\leq \chi_{m}\ll 1$ and diamagnetic ones with $-1\ll \chi_{m}\leq 0$, which all have a typical linear relation between $M\left(r,t\right)$ and $H\left(r,t\right)$.

Typical values of the magnetic susceptibility/relative permittivity for representative substances are presented in Appendix B, together with the basics of magnetic polarization [26,41,42]. Practically, all typical diamagnets and paramagnets have $\left|\chi_{m}\right|$ well below 10^−2^ so that the ‘M-H, χ_m_’ formulation obeys the above convergence criterion, $|-\chi_{m}N|<1$, in the entire range 0≤N≤1.

Superconductors can also be included in the discussion since they are diamagnets; ultimately, in a small part of the H-T phase diagram, they exhibit the perfect diamagnetic behavior with $\mu_{r}=0$, else $\chi_{m}=-1$ (Meissner state) [43,44,45]. Thus, even in this category of materials, the above convergence criterion holds (though marginally in the Meissner state).

### 2.3. Susceptibility and Polarization in Realistic Specimens of LHI Dielectric Materials: The ‘P-D, χ_ε_’ Formulation

Recently, a new aspect of the susceptibility and polarization in the wide class of LHI dielectric materials was introduced in [21]. According to the so-called ‘P-D, χ_ε_’ formulation [21], the susceptibility $\chi_{\varepsilon }$, with $-1\leq \chi_{\varepsilon }\leq 0$, couples linearly the reverse polarization, $\tilde{P}=-P$, with the electric displacement, D, in a *direct* way, $\tilde{P}=\chi_{\varepsilon }D$. In [21], the ‘P-D, χ_ε_’ formulation was introduced in electrostatics. Here, the time-dependent case is exploited. By taking into account the depolarizing factor, it is shown that the susceptibility and polarization of the LHI dielectric and magnetic materials can be described on a common basis, according to the ‘P-D, χ_ε_’ and ‘M-H, χ_m_’ formulations, respectively. Importantly, the ‘P-D, χ_ε_’ formulation is immune to the conceptual and mathematical discrepancies of the standard ‘P-E, χ_e_’ formulation discussed above.

To address these issues, the series scheme already used above for the cases of the ‘P-E, χ_e_’ and ‘M-H, χ_m_’ formulations is employed. However, instead of the electric field, $E\left(r,t\right)$, investigated in Section 2.1 for the ‘P-E, χ_e_’ formulation, here, we focus on the electric displacement, $D\left(r,t\right)$, since this is the primary vector field in the ‘P-D, χ_ε_’ aspect [21]. Accordingly, the system under investigation refers to a dielectric specimen of limited size, in free space, subjected to a uniform *external* electric displacement, $D_{ext}\left(r,t\right)=D_{0}f(\omega t)\hat{z}$, where f(ωt) is a harmonic function of time and $D_{0}=\varepsilon_{0}E_{0}$ ($D_{ext}\left(r,t\right)=\varepsilon_{0}E_{ext}\left(r,t\right)$). Again, the frequency, ω, is low, so the quasi-static limit applies. Since the methodology is the same as the one addressed above in detail for the ‘P-E, χ_e_’ and ‘M-H, χ_m_’ cases, the algebraic part will be reviewed relatively briefly.

**Figure 3:**
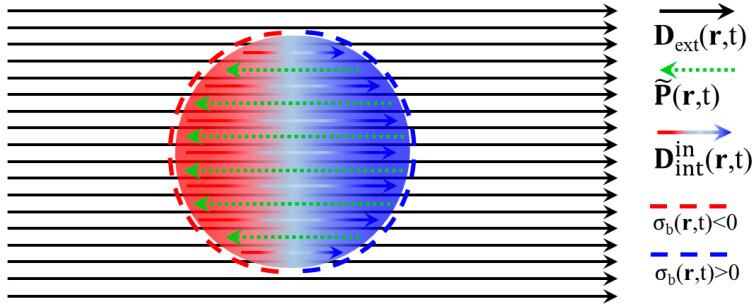
Schematic illustration of a simple spherical specimen of an LHI dielectric material subjected to an *external* electric displacement field, $D_{ext}\left(r,t\right)=D_{0}f(\omega t)\hat{z}$, according to the ‘P-D, χ_ε_’ formulation. The illustration focuses on the processes occurring at the interior of the specimen, so the dipolar *internal* electric displacement field at the exterior of the specimen, $D_{int}^{out}\left(r,t\right)$, is not shown. The color scale illustrates the spatial variation of the *internal* electric *free* scalar potential, $U_{f,int}^{in}\left(r\right)=(N\chi_{\varepsilon }/(1+N\chi_{\varepsilon }))E_{0}z$, which produces $D_{int}^{in}\left(r,t\right)$ through $D_{int}^{in}\left(r,t\right)=-\varepsilon_{0}\nabla \cdot U_{f,int}^{in}\left(r,t\right)$ (see [21] and relation (85)).

The discussion of this Section 2.3 is based on the schematic illustration of Figure 3; the specimen is the same dielectric sphere already discussed from the viewpoint of the ‘P-E, χ_e_’ formulation in Section 2.1. The sphere exhibits homogeneous reverse polarization $\tilde{P}\left(r,t\right)=-P\left(r,t\right),$ which develops at its interior upon application of the uniform $D_{ext}\left(r,t\right)=D_{0}f(\omega t)\hat{z}$ (see below and [21]). Also, as shown below, even for the ‘P-D, χ_ε_’ formulation discussed here, the respective depolarizing factor, N, can be calculated analytically. The homogeneous reverse polarization, $\tilde{P}\left(r,t\right)=-P\left(r,t\right)$, which ultimately is established at the interior of the specimen, is discontinuous at its surface, so that only surface *bound* charges exist with density $\sigma_{b}\left(r,t\right){|}_{S}=-\hat{n}\cdot \tilde{P}\left(r,t\right){|}_{S}$ (see below and [21]). These produce the *internal* electric displacement field, $D_{int}^{in}\left(r,t\right)$, which adds to $D_{ext}\left(r,t\right)$, applied by the user, to ultimately establish the total electric displacement field $D^{in}\left(r,t\right)=D_{ext}^{in}\left(r,t\right)+D_{int}^{in}\left(r,t\right)$ at the interior of the specimen. Below, it will be shown that $D_{int}^{in}\left(r,t\right)$ is parallel/anti-parallel to $P\left(r,t\right)$/$\tilde{P}\left(r,t\right)$ and parallel to $D_{ext}^{in}\left(r,t\right)$. The illustration focuses on the processes occurring at the interior of the specimen, so the dipolar *internal* electric displacement field at the exterior of the specimen, $D_{int}^{out}\left(r,t\right)$, is not shown.

#### 2.3.1. Standard Calculations for the ‘P-D, χ_ε_’ Formulation

In the ‘P-D, χ_ε_’ formulation, it is the *external* electric displacement, $D_{ext}(r,t)$, applied by the user, which controls the reverse polarization of the specimen $\tilde{P}\left(r,t\right)=-P\left(r,t\right)$. At the permanent state, the reverse polarization should be [21].

(80) $$\tilde{P}\left(r,t\right)=\chi_{\varepsilon }D^{in}\left(r,t\right)$$

where $D^{in}\left(r,t\right)$ is the total electric displacement at the interior of the specimen. Obviously

(81) $$D^{in}\left(r,t\right)=D_{ext}^{in}\left(r,t\right)+D_{int}^{in}\left(r,t\right)$$

where $D_{int}^{in}\left(r\right)$ is the *internal* component produced by the surface *bound* charges of density $\sigma_{b}\left(r,t\right){|}_{S}=-\hat{n}\cdot \tilde{P}\left(r,t\right){|}_{S}$ where $\hat{n}$ is the unit vector with direction from the interior to the exterior of the dielectric specimen. The *internal* electric displacement, $D_{int}^{in}\left(r,t\right)$, should be anti-parallel to the reverse polarization, $\tilde{P}\left(r,t\right)$, through

(82) $$D_{int}^{in}\left(r,t\right)=-N\tilde{P}\left(r,t\right)$$

where N is the so-called ‘P-D’ depolarizing factor, a scalar quantity. Currently, there is no knowledge on the nature of N for the ‘P-D, χ_ε_’ formulation. In Section 3, below, it is documented that the depolarizing factor of the ‘P-D, χ_ε_’ formulation (i) ranges within 0≤N≤1, and (ii) is different from the respective factor of the ‘P-E, χ_e_’ formulation.

By combining the above relations (80)–(82), the following relation is obtained

(83) $$\tilde{P}\left(r,t\right)=\left(\frac{\chi_{\varepsilon }}{1+N\chi_{\varepsilon }}\right)D_{ext}^{in}\left(r,t\right)$$

The dimensionless proportionality factor between $\tilde{P}\left(r,t\right)$ and $D_{ext}^{in}\left(r,t\right)$ can be termed ‘P-D’ *extrinsic* electric susceptibility

(84) $$\chi_{\varepsilon ,extr}=\frac{\chi_{\varepsilon }}{1+N\chi_{\varepsilon }}$$

While it should be recalled that $\chi_{\varepsilon }$ is the ‘P-D’ *intrinsic* electric susceptibility ($\chi_{\varepsilon }\equiv \chi_{\varepsilon ,intr}$), a quantity representative of the endogenous dielectric properties of the parent LHI material (not just of the specific specimen under investigation). Accordingly, $\chi_{\varepsilon ,extr}$ is a quantity which is controlled by the ‘P-D’ depolarizing factor, N, thus representing the exogenous properties of the particular specimen under investigation. Recall that N depends on (i) the shape of the specimen, (ii) the relative dimensions of the specimen, and (iii) the relative orientation between the specimen and the *external* applied field [22,23,24,25,26]. By combining the above relations (82) and (83), at the interior of the specimen for the *internal* electric displacement, the following relation is obtained

(85) $$D_{int}^{in}\left(r,t\right)=-\left(\frac{N\chi_{\varepsilon }}{1+N\chi_{\varepsilon }}\right)D_{ext}^{in}\left(r,t\right)$$

From this relation, it is concluded that $D_{int}^{in}\left(r,t\right)$ is parallel to $D_{ext}^{in}\left(r,t\right)$ (notice that by definition 0≤N≤1 and $-1\leq \chi_{\varepsilon }\leq 0$).

Finally, by using the above relations (81) and (85), for the total electric displacement $D^{in}\left(r,t\right)$, at the interior of the specimen, the following relation is obtained

(86) $$D^{in}\left(r,t\right)=\left(\frac{1}{1+N\chi_{\varepsilon }}\right)D_{ext}^{in}\left(r,t\right)$$

The factor $1/(1+N\chi_{\varepsilon })$ which appears in the above relations (83)–(86), can be written as the sum of an infinite geometric series [34]

(87) $$\sum_{i=0}^{\infty }{\left(-{N\chi }_{\varepsilon }\right)}^{i}=\frac{1}{1+N\chi_{\varepsilon }}$$

Under this substitution, relations (83)–(86) transform to the respective ones

(88) $$\tilde{P}\left(r,t\right)=\left(\sum_{i=0}^{\infty }{\left(-{N\chi }_{\varepsilon }\right)}^{i}\right)\chi_{\varepsilon }D_{ext}^{in}\left(r,t\right)$$

(89) $$\chi_{\varepsilon ,extr}=\chi_{\varepsilon }\left(\sum_{i=0}^{\infty }{\left(-{N\chi }_{\varepsilon }\right)}^{i}\right)$$

(90) $$D_{int}^{in}\left(r,t\right)=-N\left(\sum_{i=0}^{\infty }{\left(-{N\chi }_{\varepsilon }\right)}^{i}\right)\chi_{\varepsilon }D_{ext}^{in}\left(r,t\right)$$

and

(91) $$D^{in}\left(r,t\right)=\left(\sum_{i=0}^{\infty }{\left(-{N\chi }_{\varepsilon }\right)}^{i}\right)D_{ext}^{in}\left(r,t\right)$$

Formally speaking, the above relations are meaningful only when the geometric series converges, that is, only when the following condition holds [34]

(92) $$|-N\chi_{\varepsilon }|<1$$

Recall that in the ‘P-D, χ_ε_’ formulation, by definition, the electric susceptibility, $\chi_{\varepsilon }$, ranges within $-1\leq \chi_{\varepsilon }\leq 0$ [21]. Also, below, in Section 3, it is shown that the depolarizing factor, N, ranges within 0≤N≤1. *Accordingly, the above convergence condition is inherently satisfied in the ‘P-D, χ_ε_’ formulation.* This is a fundamental difference with the ‘P-E, χ_e_’ formulation, which suffers from the endogenous drawback of non-compliance with the relevant convergence condition of relation (46), $|-N\chi_{e}|<1$. This drawback stems from the fact that, though the ‘P-E’ depolarizing factor ranges within 0≤N≤1 (in consistency with that of the ‘P-D’ one), the ‘P-E’ susceptibility ranges within $0\leq \chi_{e}<\infty$. Thus, while in the ‘P-E, χ_e_’ formulation, the condition $|-N\chi_{e}|<1$ is not inherently satisfied, in the ‘P-D, χ_ε_’ one, it is satisfied *by default*.

For the sake of completeness, below, the series scheme is employed for the ‘P-D, χ_ε_’ formulation. It is clearly shown that the above relations can be obtained in the framework of the polarization cascade discussed for both the ‘P-E, χ_e_’ and ‘M-H, χ_m_’ formulations in the above Section 2.1.2, Section 2.1.3, Section 2.1.4 and Section 2.2.2, Section 2.2.3, Section 2.2.4, respectively.

#### 2.3.2. Electric Displacement, D(r,t), Based on a Scheme of Series

Following the scheme of series, the user-applied $D_{ext}(r,t)$ can be considered as the zeroth-order term, $D_{0}^{in}\left(r,t\right)$, of the total electric displacement, $D^{in}\left(r,t\right)$, which gradually develops during the transient stage and ultimately will be established at the steady state at the interior of the dielectric specimen. The zeroth-order term, $D_{0}^{in}\left(r,t\right)$, will polarize, partially, the specimen by inducing a zeroth-order term of the reverse polarization, ${\tilde{P}}_{0}\left(r,t\right)$, through ${\tilde{P}}_{0}\left(r,t\right)=\chi_{\varepsilon }D_{0}^{in}\left(r,t\right)$. In general, the reverse polarization, $\tilde{P}\left(r,t\right)$, of a uniformly polarized dielectric specimen (as the spherical one studied here), is accompanied by only a surface density of *bound* charges, $\sigma_{b}\left(r,t\right){|}_{S}=-\hat{n}\cdot \tilde{P}\left(r,t\right){|}_{S}$. The latter acts as a secondary source and produces an *internal* electric displacement $D_{int}^{in}\left(r,t\right)=-N\tilde{P}\left(r,t\right)$ at the interior of the dielectric specimen. Accordingly, in our case, the zeroth-order term of the reverse polarization ${\tilde{P}}_{0}\left(r,t\right)$ will produce a higher-order term, that is, the first-order term, of the *internal* electric displacement through $D_{int,1}^{in}\left(r,t\right)=-N{\tilde{P}}_{0}\left(r,t\right)$. The first-order term, $D_{int,1}^{in}\left(r,t\right)$, will induce the first-order term in the reverse polarization through ${\tilde{P}}_{1}\left(r,t\right)=\chi_{\varepsilon }D_{int,1}^{in}\left(r,t\right)$. The latter will subsequently produce a second-order term in the *internal* electric displacement through $D_{int,2}^{in}\left(r,t\right)=-N{\tilde{P}}_{1}\left(r,t\right),$ and so on. Generalizing this scheme, the (i-1)-order term of the induced reverse polarization should be ${\tilde{P}}_{i-1}\left(r,t\right)=\chi_{\varepsilon }D_{int,i-1}^{in}\left(r,t\right)$, whereas the (i)-order term of the *internal* electric displacement should be $D_{int,i}^{in}\left(r,t\right)=-N{\tilde{P}}_{i-1}\left(r,t\right)$. The engagement of the latter relations on ${\tilde{P}}_{i-1}\left(r,t\right)$ and $D_{int,i}^{in}\left(r,t\right)$ in a recursive way enables us to obtain the following relation.

(93) $$D_{int,i}^{in}\left(r,t\right)=\left(-{N\chi }_{\varepsilon }\right)D_{int,i-1}^{in}\left(r,t\right)=\left(-{N\chi }_{\varepsilon }\right)\left(-{N\chi }_{\varepsilon }\right)D_{int,i-2}^{in}\left(r,t\right)=\;\left(-{N\chi }_{\varepsilon }\right)\left(-{N\chi }_{\varepsilon }\right)\left(-{N\chi }_{\varepsilon }\right)D_{int,i-3}^{in}\left(r,t\right)=\ldots$$

else

(94) $$D_{int,i}^{in}\left(r,t\right)={\left(-{N\chi }_{\varepsilon }\right)}^{i}D_{0}^{in}\left(r,t\right)$$

Now it is easily seen that the total electric displacement, $D^{in}\left(r,t\right)$, at the interior of the dielectric specimen is described by an infinite series as follows:

(95) $$D^{in}\left(r,t\right)=D_{0}^{in}\left(r,t\right)+D_{int,1}^{in}\left(r,t\right)+D_{int,2}^{in}\left(r,t\right)+\ldots +D_{int,i}^{in}\left(r,t\right)+\ldots \;={\left(-{N\chi }_{\varepsilon }\right)}^{0}D_{0}^{in}\left(r,t\right)+{\left(-{N\chi }_{\varepsilon }\right)}^{1}D_{0}^{in}\left(r,t\right)+{\left(-{N\chi }_{\varepsilon }\right)}^{2}D_{0}^{in}\left(r,t\right)+\ldots +{\left(-{N\chi }_{\varepsilon }\right)}^{i}D_{0}^{in}\left(r,t\right)+\ldots$$

else

(96) $$D^{in}\left(r,t\right)=\left(\sum_{i=0}^{\infty }{\left(-{N\chi }_{\varepsilon }\right)}^{i}\right)D_{0}^{in}\left(r,t\right)$$

where recall that $D_{0}^{in}\left(r,t\right)=D_{ext}\left(r,t\right)$.

As routinely discussed many times above, this geometric series converges only when the following condition holds, $|-N\chi_{\varepsilon }|<1$, resulting in $\sum_{i=0}^{\infty }{\left(-{N\chi }_{\varepsilon }\right)}^{i}=1/\left(1+N\chi_{\varepsilon }\right)$. Under these circumstances, the total electric displacement at the interior of the specimen will be given by

(97) $$D^{in}\left(r,t\right)=\left(\frac{1}{1+N\chi_{\varepsilon }}\right)D_{ext}\left(r,t\right)$$

while the *internal* electric displacement at the same area will simply be given by $D_{int}^{in}\left(r,t\right)=D^{in}\left(r,t\right)-D_{ext}^{in}\left(r,t\right)$, thus

(98) $$D_{int}^{in}\left(r,t\right)=-\left(\frac{N\chi_{\varepsilon }}{1+N\chi_{\varepsilon }}\right)D_{ext}\left(r,t\right)$$

These results, relations (97) and (98), are identical to the ones obtained previously, relations (86) and (85), respectively, since $D_{ext}\left(r,t\right)=D_{ext}^{in}\left(r,t\right)$.

#### 2.3.3. Reverse Polarization, $\tilde{P}(r,t)$, Based on a Scheme of Series

The recursive sequence described above can be applied to the reverse polarization, as well, as shown below. Accordingly, $\tilde{P}\left(r,t\right),$ which only exists at the interior of the specimen, can be described by an infinite series. The zeroth-order term of $\tilde{P}\left(r,t\right)$ will be induced by the zeroth-order term of the electric displacement, $D_{0}^{in}\left(r,t\right)$, that is, the *external* electric displacement, $D_{ext}\left(r,t\right)$, through ${\tilde{P}}_{0}\left(r,t\right)=\chi_{\varepsilon }D_{0}^{in}\left(r,t\right)=\chi_{\varepsilon }D_{ext}\left(r,t\right)$. It can easily be shown that, in general, the (i)-order term will be

(99) $${\tilde{P}}_{i}\left(r,t\right)={\left(-{N\chi }_{\varepsilon }\right)}^{i}\chi_{\varepsilon }D_{ext}\left(r,t\right)$$

Accordingly, the reverse polarization is given by

(100) $$\tilde{P}\left(r,t\right)=\sum_{i=0}^{\infty }{\tilde{P}}_{i}\left(r,t\right)=\left(\sum_{i=0}^{\infty }{\left(-{N\chi }_{\varepsilon }\right)}^{i}\right)\chi_{\varepsilon }D_{ext}\left(r,t\right)$$

As discussed above, this geometric series converges only when the condition $|-N\chi_{\varepsilon }|<1$ is satisfied. Then, relation (100) takes the form

(101) $$\tilde{P}\left(r,t\right)=\left(\frac{\chi_{\varepsilon }}{1+N\chi_{\varepsilon }}\right)D_{ext}\left(r,t\right)$$

In the above relations, it was recalled that $D_{ext}\left(r,t\right)=D_{ext}^{in}\left(r,t\right)$. Also, in relation (101), the dimensionless proportionality factor of the two vector fields (i.e., excluding $\varepsilon_{0}$) is the so-called *extrinsic* electric susceptibility

(102) $$\chi_{\varepsilon ,extr}=\frac{\chi_{\varepsilon }}{1+N\chi_{\varepsilon }}$$

with the physical meaning already assigned in the above discussions. Finally, as already shown above, $D^{in}\left(r,t\right)=D_{ext}\left(r,t\right)/(1+N\chi_{\varepsilon }),$ so that the above relation (101) takes the equivalent form.

(103) $$\tilde{P}\left(r,t\right)=\chi_{\varepsilon }D^{in}\left(r,t\right)$$

This is the relation that formally defines the reverse polarization, $\tilde{P}\left(r,t\right)$, in respect to the total electric displacement, $D^{in}\left(r,t\right)$, at the interior of the specimen, according to the ‘P-D, χ_ε_’ formulation [21].

#### 2.3.4. Bound Surface Charge Density, $\sigma_{b}\left(r,t\right){|}_{S}$, Based on a Scheme of Series for the ‘P-D, χ_ε_’ Formulation

Here, the recursive sequence is applied to the *bound* surface charge density, $\sigma_{b}\left(r,t\right){|}_{S}$, which gradually develops at the transient stage and eventually establishes at the steady state at the surface of the dielectric specimen. It is noted that in the ‘P-D, χ_ε_’ formulation, $\sigma_{b}\left(r,t\right){|}_{S}$ is given through

(104) $$\sigma_{b}\left(r,t\right){|}_{S}=\hat{n}\cdot P\left(r,t\right){|}_{S}=-\hat{n}\cdot \tilde{P}\left(r,t\right){|}_{S}$$

The *bound* charge density, $\sigma_{b}\left(r,t\right){|}_{S}$, is induced by the primary source, the external electric displacement, $D_{ext}(r,t)$, that stems from the *free* charge density. Then, $\sigma_{b}\left(r,t\right){|}_{S}$ acts as a secondary source, thus producing the *internal* electric displacement, $D_{int}^{in}\left(r,t\right)$. The zeroth-order term of the reverse polarization, ${\tilde{P}}_{0}\left(r,t\right)=\chi_{\varepsilon }D_{0}^{in}\left(r,t\right)$, induces a zeroth-order term in the *bound* surface charge density, $\sigma_{b,0}\left(r,t\right){|}_{S}=-\hat{n}\cdot {\tilde{P}}_{0}\left(r,t\right){|}_{S}=-\hat{n}\cdot (\chi_{\varepsilon }D_{0}^{in}\left(r,t\right)){|}_{S}$. Then, $\sigma_{b,0}\left(r,t\right){|}_{S}$ will produce a first-order term in the *internal* electric displacement, $D_{int,1}^{in}\left(r,t\right)$, while $D_{int,1}^{in}\left(r,t\right)$ will induce a first-order term in the reverse polarization, ${\tilde{P}}_{1}\left(r,t\right)$, which will add a first-order term in the *bound* surface charge density, $\sigma_{b,1}\left(r,t\right){|}_{S}=-\hat{n}\cdot {\tilde{P}}_{1}\left(r,t\right){|}_{S}$. The latter produces the second-order term of the *internal* electric displacement, $D_{int,2}^{in}\left(r,t\right)$, and so on, until the steady state is established. It is concluded that the (i)-order term of the *bound* surface charge density is given by $\sigma_{b,i}\left(r,t\right){|}_{S}=-\hat{n}\cdot {\tilde{P}}_{i}\left(r,t\right){|}_{S}$, while the (i)-order term of the reverse polarization has the form ${\tilde{P}}_{i}\left(r,t\right)={\left(-N\chi_{\varepsilon }\right)}^{i}\chi_{\varepsilon }D_{0}^{in}\left(r,t\right)$. Thus, the (i)-order term of the *bound* surface charge density is $\sigma_{b,i}\left(r,t\right){|}_{S}=-{\left(-N\chi_{\varepsilon }\right)}^{i}\chi_{\varepsilon }\hat{n}\cdot D_{0}^{in}\left(r,t\right){|}_{S}$. Finally, at the steady state, the complete *bound* surface charge density at the interface of the specimen and the free space is

(105) $$\sigma_{b}\left(r,t\right){|}_{S}=\sum_{i=0}^{\infty }\sigma_{b,i}\left(r,t\right){|}_{S}=-\left(\sum_{i=0}^{\infty }{\left(-N\chi_{\varepsilon }\right)}^{i}\right)\chi_{\varepsilon }\hat{n}\cdot D_{0}^{in}\left(r,t\right){|}_{S}$$

else

(106) $$\sigma_{b}\left(r,t\right){|}_{S}=-\left(\frac{\chi_{\varepsilon }}{1+N\chi_{\varepsilon }}\right)\hat{n}\cdot D_{ext}\left(r,t\right){|}_{S}$$

a relation which holds under the assumption that the convergence condition $|-N\chi_{\varepsilon }|<1$ is satisfied. The exact same result can be obtained by other means. The *internal* electric displacement produced by $\sigma_{b}\left(r,t\right){|}_{S}$ is $D_{int}^{in}\left(r,t\right)=-(N\chi_{\varepsilon }/(1+N\chi_{\varepsilon }))D_{ext}(r,t)$. As expected, $D_{int}^{in}\left(r,t\right)$ is parallel to $D_{ext}\left(r,t\right)$ and relates to the reverse polarization through $D_{int}^{in}\left(r,t\right)=-N\tilde{P}\left(r,t\right)$. Finally, by recalling relation (28), we realize that in reference to the ‘P-D, χ_ε_’ formulation, the reverse *bound* charge surface density can be defined through

(107) $${\tilde{\sigma }}_{b}\left(r,t\right){|}_{S}=-\sigma_{b}\left(r,t\right){|}_{S}=\left(\frac{\chi_{\varepsilon }}{1+N\chi_{\varepsilon }}\right)\hat{n}\cdot D_{ext}\left(r,t\right){|}_{S}$$

Since this expression is suitable to reveal symmetry arguments in a concise way (see below). Recall that in the above relations (106) and (107), $D_{ext}\left(r,t\right)=D_{ext}^{in}\left(r,t\right)$.

Summarizing the processes described in the above Section 2.3.2, Section 2.3.3 and Section 2.3.4 for the ‘P-D, χ_ε_’ formulation, the *external* electric displacement $D_{ext}\left(r,t\right)$ induces a reverse polarization $\tilde{P}\left(r,t\right)=\left(\chi_{\varepsilon }/\left(1+N\chi_{\varepsilon }\right)\right)D_{ext}\left(r,t\right)$ at the dielectric specimen. In turn, the discontinuity of $\tilde{P}\left(r,t\right)$ at the surface of the specimen establishes a reverse *bound* surface charge density ${\tilde{\sigma }}_{b}\left(r,t\right){|}_{S}=-\sigma_{b}\left(r,t\right){|}_{S}=(\chi_{\varepsilon }/(1+N\chi_{\varepsilon }))\hat{n}\cdot D_{ext}(r,t){|}_{S},$ which produces an *internal* electric displacement $D_{int}^{in}\left(r,t\right)=-(N\chi_{\varepsilon }/(1+N\chi_{\varepsilon }))D_{ext}(r,t)$. The latter is inherently parallel to the *external* one, $D_{ext}\left(r,t\right)$. Accordingly, the *external* electric displacement, $D_{ext}\left(r,t\right)$, inside the dielectric specimen evolves to $D^{in}\left(r,t\right)=(1/(1+N\chi_{\varepsilon }))D_{ext}(r,t)$. Since the *internal* electric displacement, $D_{int}^{in}\left(r,t\right)$, is parallel to the *external* one, at the interior of the specimen, the relation $D^{in}\left(r,t\right)>D_{ext}\left(r,t\right)$, always holds. Accordingly, $D_{int}^{in}\left(r,t\right)$ clearly assists the polarization of the dielectric specimen, in contrast to $E_{int}^{in}\left(r,t\right),$ which acts towards its depolarization.

#### 2.3.5. Compliance of the ‘P-D, χ_ε_’ Formulation with the Convergence Condition of the Series

From the argumentation and the algebra presented here in the entire Section 2.3, it is easily seen that the ‘P-D, χ_ε_’ formulation restores the two basic drawbacks of the standard ‘P-E, χ_e_’ formulation: the conceptually misleading causality between $P\left(r,t\right)$ and $E\left(r,t\right)$ and the serious algebraic hurdle of non-compliance with basic convergence conditions. Referring to the latter, recall that in the ‘P-E, χ_e_’ formulation, the depolarizing factor ranges within 0≤N≤1, while the ‘P-E’ susceptibility ranges within $0\leq \chi_{e}<\infty$. Thus, in the ‘P-E, χ_e_’ formulation, the convergence condition $|-N\chi_{e}|<1$ is not inherently satisfied over the entire range of values. Instead, either $\chi_{e}$ should be limited within $0\leq \chi_{e}<1/N$ (given that the depolarizing factor is fixed by the exogenous characteristics of each particular specimen), or N should range within $0\leq N<1/\chi_{e}$ (given that the intrinsic susceptibility is fixed by the endogenous characteristics of the parent LHI material). In the ‘P-D, χ_ε_’ formulation, the condition $|-N\chi_{\varepsilon }|<1$ is satisfied *by default* due to the fact that $-1\leq \chi_{\varepsilon }\leq 0$ and 0≤N≤1.

## 3. Endogenous Symmetry and Equivalence of the ‘P-E, χ_e_’ and ‘P-D, χ_ε_’ Formulations

The newly introduced ‘P-D, χ_ε_’ formulation should give results absolutely equivalent to the ones of the standard ‘P-E, χ_e_’ formulation. To assess this expectation, we compare the results obtained above; all relations of the electric field obtained by using the ‘P-E, χ_e_’ formulation are compared to the ones of the electric displacement field obtained by means of the ‘P-D, χ_ε_’ formulation. To this effect, in Table 1 below, for convenience, the solutions found with each formulation are summarized. In some cases, simple rearrangements of factors/constants have been performed to facilitate the comparison and the respective discussion. The implications of this comparison are both unexpected and remarkable.

The symmetry between the expressions of the vector fields for the two formulations is obvious; $E^{in}\left(r,t\right)$, $E_{int}^{in}\left(r,t\right)$ and $P\left(r,t\right)$ for the ‘P-E, χ_e_’ formulation and $D^{in}\left(r,t\right)$, $D_{int}^{in}\left(r,t\right)$ and $\tilde{P}\left(r,t\right)$ for the ‘P-D, χ_ε_’ one, acquire the exact same form of relations in reference to the external stimulus, $E_{ext}\left(r,t\right)$ and $D_{ext}\left(r,t\right)$, respectively. Also, the scalar entities, $\sigma_{b}\left(r,t\right){|}_{S}$ and ${\tilde{\sigma }}_{b}\left(r,t\right){|}_{S}$ acquire the exact same form in the ‘P-E, χ_e_’ and ‘P-D, χ_ε_’ formulation, respectively.

Most importantly, the ‘P-D, χ_ε_’ formulation should give consistent results with the ‘P-E, χ_e_’ one. Accordingly, the pairs of relations of Table 1 are compared by using relation (18), as well. To this effect, $E_{ext}\left(r,t\right)=E_{0}f\left(\omega t\right)\hat{z}$ and $D_{ext}\left(r,t\right)=D_{0}f\left(\omega t\right)\hat{z}$ (where obviously $D_{0}=\varepsilon_{0}E_{0}$) should be used in all four comparisons. It is concluded, in a consistent way, that in all four cases *the depolarizing factor,* N*, cannot be the same in the ‘P-E, χ_e_’ and ‘P-D, χ_ε_’ formulations, else their equivalence is not satisfied*. To resolve this issue, it should be assumed that the depolarizing factor is different in the ‘P-E, χ_e_’ and ‘P-D, χ_ε_’ formulations, namely $N_{e}$ and $N_{\varepsilon }$, respectively. Then, equating once again each pair of relations, the same conclusion is obtained in a consistent way; in all four cases, the following relation should hold:

(108) $$N_{e}+N_{\varepsilon }=1$$

This result is surprising and intriguing. First, it is noted that the depolarizing factors of the two formulations have a ‘complementary character’ since their sum is equal to unity. Second, this fact is consistent with the definition of a depolarizing factor, N, since under all circumstances the sum of Ns of different origin (e.g., along the different principal axes of an ellipsoid) should be equal to unity. Third, since $N_{e}$ ranges within $0\leq N_{e}\leq 1$, it is ensured that $N_{\varepsilon }$ ranges within $0\leq N_{\varepsilon }\leq 1$, as well. Fortunately, this guarantees that the convergence criterion $|-N_{\varepsilon }\chi_{\varepsilon }|<1$ is inherently fulfilled in the ‘P-D, χ_ε_’ formulation (see the discussion in Section 2.3, above).

The above general outcome can be verified against some reference problems encountered in many applications. Table 2 shows some representative examples coming from all three coordinate systems: Cartesian, cylindrical, and spherical. The presented systems refer to dielectric specimens subjected to a static, *external* electric field originating from *free* charges (left column). All dielectric specimens originate from an LHI parent material of *intrinsic* electric susceptibility $\chi_{e}$ and $\chi_{\varepsilon }$, according to the ‘P-E’ and ‘P-D’ formulation, respectively. Here we focus exclusively on the polarization, $P\left(r\right)$, and the reverse polarization, $\tilde{P}\left(r\right)$, at the interior of the specimen and present the as-obtained solutions (top of each cell) for both formulations, ‘P-E’ (middle column) and ‘P-D’ (right column), respectively. These as-obtained solutions on $P\left(r\right)$ and $\tilde{P}\left(r\right)$ were reported in [21] (detailed solutions on the electric field, $E\left(r\right)$, displacement, $D\left(r\right)$, and *bound* charge surface density, $\sigma_{b}\left(r,t\right){|}_{S}$, can be found in [21], as well). Most importantly, to serve the purposes of our present study, for each case in Table 2, a reformed version of the respective as-obtained solution is also presented (bottom of each cell). In this equivalent version, the original solution is rewritten so that the presence of the respective depolarizing factor, $N_{e}$ for the ‘P-E, χ_e_’ formulation and $N_{\varepsilon }$ for the ‘P-D, χ_ε_’ one, is clearly evidenced.

From the reformed version of the solutions, in all five cases, it becomes clear that $P\left(r\right)$ and $\tilde{P}\left(r\right)$ relate consistently to the relevant *external* field, $E\left(r\right)$ and $D\left(r\right)$, of the ‘P-E, χ_e_’ and ‘P-D, χ_ε_’, respectively, through the respective *extrinsic* electric susceptibility, $\chi_{e,extr}=\chi_{e}/\left(1+N_{e}\chi_{e}\right)$ (relation (32)) and $\chi_{\varepsilon ,extr}=\chi_{\varepsilon }/\left(1+N_{\varepsilon }\chi_{\varepsilon }\right)$ (relation (84)), as it should. Most importantly, it can be easily seen that in each pair of all five cases presented here, the depolarizing factors satisfy relation (108), $N_{e}+N_{\varepsilon }=1$. Finally, by using the interrelation between $\chi_{e}$ and $\chi_{\varepsilon }$, relation (18), it can be easily checked that the two expressions of the polarization are equivalent, as they should be.

*The introduction of the depolarizing factor,* $N_{\varepsilon }$ *, as an inherent property of the ‘P-D, χ_ε_’ formulation, completes our aspect and makes it effective for utilization in all realistic cases where specimens of limited size are considered. This is highly important for the description of applications and constitutes one of the reasons that make the ‘P-D, χ_ε_’ formulation equally attractive, if not advantageous, to the standard ‘P-E, χ_e_’ formulation employed today.*

## 4. Maxwell’s Equations and Relevant Time-Dependent Processes

The ‘P-D, χ_ε_’ formulation was recently introduced in [21] for the case of electrostatics, where its consistency with the ‘P-E, χ_e_’ one was verified for a small number of representative examples. In Section 2 above, the quasi-static limit of low frequency was investigated. Here, the fully time-dependent case is clarified, without restriction in the frequency. An important property of the ‘P-D, χ_ε_’ formulation is clearly proved; it leaves Maxwell’s equations unaltered. Recall that in the ‘P-E, χ_e_’ formulation, an LHI medium is characterized by a susceptibility $\chi_{e}$ ($0\leq \chi_{e}<\infty$), relative permittivity $\varepsilon_{r}=1+\chi_{e}$ ($1\leq \varepsilon_{r}<\infty$), and permittivity $\varepsilon =\varepsilon_{r}\varepsilon_{0}$ [8,9,10,11,12,13,14,15]. On the other hand, the ‘P-D, χ_ε_’ formulation introduces the susceptibility $\chi_{\varepsilon }$ ($-1\leq \chi_{\varepsilon }\leq 0$), relative permittivity $\varepsilon_{r\varepsilon }={\left(1+\chi_{\varepsilon }\right)}^{-1}$ ($1\leq \varepsilon_{r\varepsilon }<\infty$) and permittivity $\varepsilon_{\varepsilon }=\varepsilon_{r\varepsilon }\varepsilon_{0}$ [21]. In the advent of any magnetic properties, these are expressed through a susceptibility, $\chi_{m}$, relative permeability, $\mu_{r}=1+\chi_{m}$, and permeability, $\mu =\mu_{0}\mu_{r}$. By using relation (18), the relevant definition, $\varepsilon_{r}=1+\chi_{e}$, of the ‘P-E, χ_e_’ formulation [8,9,10,11,12,13,14,15], and the respective one, $\varepsilon_{r\varepsilon }={\left(1+\chi_{\varepsilon }\right)}^{-1}$, of the ‘P-D, χ_ε_’ formulation [21], it is easily seen that the two permittivities are identical, $\varepsilon_{r}=\varepsilon_{r\varepsilon }$. This important fact guarantees that Maxwell’s equations are unaltered by the ‘P-D, χ_ε_’ formulation. This should hold whether the ‘microscopic’ form of relations (1)–(4) or the ‘macroscopic’ form of relations (5)–(8) is employed. Recall that the first set of equations depends on the *primary* fields, $E\left(r,t\right)$ and $B\left(r,t\right)$, while the second one is based on the *secondary/auxiliary* fields $D\left(r,t\right)$ and $H\left(r,t\right)$ [8,9,10,11,12,13,14,15]. This fact is verified below.

First, Gauss’s law for electricity: Its ‘microscopic’ version of relation (1) depends on the total charge density, $\rho \left(r,t\right)$. The latter consists of the *free* and *bound* components through

(109) $$\rho \left(r,t\right)=\rho_{f}\left(r,t\right)+\rho_{b}\left(r,t\right)$$

In the standard ‘P-E, χ_e_’ formulation $\rho_{b}\left(r,t\right)=-\nabla \cdot P\left(r,t\right)$ so that

(110) $$\rho \left(r,t\right)=\rho_{f}\left(r,t\right)-\nabla \cdot P\left(r,t\right)$$

Then, by using relations (10), (109), and (110), relation (1) becomes

(111) $$\nabla \cdot D\left(r,t\right)=\rho_{f}\left(r,t\right)$$

which is the ‘macroscopic’ version of Gauss’s law, relation (5).

In the ‘P-D, χ_ε_’ formulation, the reverse *bound* charge volume density ${\tilde{\rho }}_{b}\left(r,t\right)=-\nabla \cdot \tilde{P}\left(r\right)=\nabla \cdot P\left(r\right)=-\rho_{b}\left(r,t\right)$, relation (27), is defined through the reverse polarization, $\tilde{P}\left(r\right)=-P\left(r,t\right)$. By using relations (10), (27), and (109), it can be easily shown that, also in the ‘P-D, χ_ε_’ formulation, the ‘microscopic’ version of Gauss’s law, relation (1), takes the ‘macroscopic’ version of relation (5).

Second, Gauss’s law for magnetism: It attains the same form in both the ‘microscopic’ and ‘macroscopic’ versions, relations (2) and (6), respectively, in both formulations, the standard ‘P-E, χ_e_’ and the alternative ‘P-D, χ_ε_’.

Third, Faraday’s law: It also attains the same form in both the ‘microscopic’ and ‘macroscopic’ versions, relations (3) and (7), respectively. In the standard ‘P-E, χ_e_’ formulation, the electric field, $E\left(r,t\right)$, is defined through the constitutive relation $E\left(r,t\right)=D\left(r,t\right)/\varepsilon_{0}\left(1+\chi_{e}\right)=D\left(r,t\right)/\varepsilon_{0}\varepsilon_{r}=D\left(r,t\right)/\varepsilon .$ In the ‘P-D, $\chi_{\varepsilon }$’ formulation, $E\left(r,t\right)$ is defined through the respective constitutive relation $E\left(r,t\right)=D\left(r,t\right)\left(1+\chi_{\varepsilon }\right)/\varepsilon_{0}=D\left(r,t\right)/\varepsilon_{0}\varepsilon_{r\varepsilon }=D\left(r,t\right)/\varepsilon_{\varepsilon }$, relation (14). By recalling relations (16)–(18) it can be easily confirmed that $\varepsilon_{r\varepsilon }=\varepsilon_{r}$ (relation (19)), else $\varepsilon_{\varepsilon }=\varepsilon$ (relation (20)). Accordingly, once $E\left(r,t\right)$ is unaltered between the ‘P-E, χ_e_’ and ‘P-D, χ_ε_’ formulations, Faraday’s law is unaltered, as well.

Fourth, Ampere’s law: Its ‘microscopic’ version, relation (4), refers to the total current density, $J\left(r,t\right)$. The latter consists of the *free*, *bound,* and *displacement* components as follows [8,9,10,11,12,13,14,15]:

(112) $$J\left(r,t\right)=J_{f}\left(r,t\right)+J_{b}\left(r,t\right)+J_{P}\left(r,t\right)$$

else

(113) $$J\left(r,t\right)=J_{f}\left(r,t\right)+\nabla \times M\left(r,t\right)+\frac{\partial P\left(r,t\right)}{\partial t}$$

where two current densities have been introduced; the density of *bound* current which relates to the magnetic polarization through $J_{b}\left(r,t\right)=\nabla \times M\left(r,t\right)$ and the density of *displacement* current which relates to the electric polarization through $J_{P}\left(r,t\right)=\partial P(r,t)/\partial t$.

In the standard ‘P-E, χ_e_’ formulation, $P\left(r,t\right)$ is expressed through relation (9) so that relation (113) takes

(114) $$J\left(r,t\right)=J_{f}\left(r,t\right)+\nabla \times M\left(r,t\right)+\chi_{e}\varepsilon_{0}\frac{\partial E\left(r,t\right)}{\partial t}$$

Then, by using relations (9), (10), (12), and (114), it can be easily shown that relation (4) becomes

(115) $$\nabla \times H\left(r,t\right)=J_{f}\left(r,t\right)+\partial D\left(r,t\right)/\partial t$$

which is the ‘macroscopic’ version of Ampere’s law, relation (8).

In our ‘P-D, χ_ε_’ formulation, the reverse polarization, $\tilde{P}\left(r\right)=-P\left(r,t\right),$ is expressed through relation (13). Accordingly, the *displacement* current of the polarization, $J_{P}\left(r,t\right)=\partial P(r,t)/\partial t$, becomes

(116) $$J_{P}\left(r,t\right)=-\chi_{\varepsilon }\frac{\partial D\left(r,t\right)}{\partial t}$$

Then, recall once again that in the ‘P-D, χ_ε_’ formulation, the relative permittivity is $\varepsilon_{r\varepsilon }={\left(1+\chi_{\varepsilon }\right)}^{-1}$ ($1\leq \varepsilon_{r\varepsilon }<\infty$), relation (16), while the permittivity is $\varepsilon_{\varepsilon }=\varepsilon_{r\varepsilon }\varepsilon_{0}$, relation (17), where $\chi_{\varepsilon }$ ($-1\leq \chi_{\varepsilon }\leq 0$) is the ‘P-D’ susceptibility. Accordingly, by using relations (12), (13), (14), (16), (17), and (113), it can be easily shown that relation (4), indeed, becomes identical to the ‘macroscopic’ version of Ampere’s law, relation (8).

Thus, Maxwell’s equations are unaltered whether we refer to the ‘P-E, χ_e_’ or the ‘P-D, χ_ε_’ formulation. This fact is crucial for the incorporation of the ‘P-D, χ_ε_’ formulation in applications that relate to time-dependent processes. For instance, let us consider the propagation of a monochromatic electromagnetic wave inside an LHI medium, which, in addition, is vacant of both charges and currents and non-dispersive. In the ‘P-E, χ_e_’ formulation, the medium is characterized by a susceptibility $\chi_{e}$ (with $0\leq \chi_{e}<\infty$), relative permittivity $\varepsilon_{r}=1+\chi_{e}$ (with $1\leq \varepsilon_{r}<\infty$), and permittivity $\varepsilon =\varepsilon_{r}\varepsilon_{0}$, while for the ‘P-D, χ_ε_’ one, the medium is subjected to the susceptibility $\chi_{\varepsilon }$ (with $-1\leq \chi_{\varepsilon }\leq 0$), relative permittivity $\varepsilon_{r\varepsilon }={\left(1+\chi_{\varepsilon }\right)}^{-1}$ (with $1\leq \varepsilon_{r\varepsilon }<\infty$), and permittivity $\varepsilon_{\varepsilon }=\varepsilon_{r\varepsilon }\varepsilon_{0}$. The magnetic properties of the LHI material (if any) are expressed through a susceptibility, $\chi_{m}$, relative permeability, $\mu_{r}=1+\chi_{m}$, and permeability, $\mu =\mu_{0}\mu_{r}$, identical in both formulations. By applying the curl in both sides of relations (3) and (4) and using standard identities, the well-known wave equation is easily obtained, identical for both $E\left(r,t\right)$ and $B\left(r,t\right)$ [12,13,14].

(117) $$\nabla^{2}E\left(r,t\right)=\varepsilon^{*}\mu \partial^{2}E\left(r,t\right)/\partial t^{2}$$

and

(118) $$\nabla^{2}B\left(r,t\right)=\varepsilon^{*}\mu \partial^{2}B\left(r,t\right)/\partial t^{2}$$

where $\varepsilon^{*}$ refers to $\varepsilon =\varepsilon_{0}\varepsilon_{r}$ or $\varepsilon_{\varepsilon }=\varepsilon_{0}\varepsilon_{r\varepsilon }$ when the ‘P-E, χ_e_’ or ‘P-D, χ_ε_’ formulation is used, respectively. The following basic entities relate closely to the propagation of the electromagnetic wave in the particular medium [12,13,14]. First, the propagation velocity, which is $u=1/\sqrt{\varepsilon^{*}\mu }$. Second, the energy density, which is defined through $U\left(r,t\right)=(1/2)(E\left(r,t\right)\cdot D\left(r,t\right)+B\left(r,t\right)\cdot H\left(r,t\right))=(1/2)(\varepsilon^{*}E^{2}\left(r,t\right)+B^{2}\left(r,t\right)/\mu )$. Third, the Poynting vector, which is given by $S\left(r,t\right)=E\left(r,t\right)\times H\left(r,t\right)=(E\left(r,t\right)\times B\left(r,t\right))/\mu$. Once both formulations, ‘P-E, χ_e_’ and ‘P-D, χ_ε_’, have identical permittivity, $\varepsilon =\varepsilon_{0}(1+\chi_{e})=\varepsilon_{0}{\left(1+\chi_{\varepsilon }\right)}^{-1}=\varepsilon_{\varepsilon }$, all physical entities that relate to the wave propagation discussed here will be identical, as well. Furthermore, the fact that $\varepsilon =\varepsilon_{\varepsilon }$ ensures that even in more complicate cases (for instance, non-monochromatic wave, dispersive medium, conducting medium, etc.), the propagation will preserve the same characteristics whether the ‘P-E, χ_e_’ or the ‘P-D, χ_ε_’ formulation is employed. The fact that the permittivities of the two formulations are the same, $\varepsilon =\varepsilon_{\varepsilon }$, also provides a simple explanation of why possible mathematical flaws of the ‘P-E, χ_e_’ formulation are neither identified experimentally nor addressed mathematically in the literature. This is due to the fact that the susceptibility, χ_e_, of the ‘P-E, χ_e_’ formulation does not enter into any of the fundamental relations of electromagnetism as a standalone physical parameter. In all cases, only the electric permittivity, ε, appears, as discussed above for Maxwell’s equations, propagation equation, etc. Notably, in both the ‘P-E, χ_e_’ and the ‘P-D, χ_ε_’ notations, the electric permittivities, $\varepsilon =\varepsilon_{0}(1+\chi_{e})$ and $\varepsilon_{\varepsilon }=\varepsilon_{0}{\left(1+\chi_{\varepsilon }\right)}^{-1}$, respectively, are identical, $\varepsilon =\varepsilon_{\varepsilon }$ (relations (18)-(20)).

*Summarizing this section, although the susceptibilities of the two formulations originate from different aspects, the specific transformation that relates them, (* $1+\chi_{\varepsilon })={\left(1+\chi_{e}\right)}^{-1}$ *(relation (18)) ensures that in the LHI materials studied here, the respective permittivities are identical,* $\varepsilon =\varepsilon_{\varepsilon }$ *, in all instances. Thus, all physical processes of electromagnetic theory depending solely on the electric permittivity will be addressed consistently, whether the ‘P-E, χ_e_’ or the ‘P-D, χ_ε_’ formulation is employed.*

## 5. Applicability, Limitations, and Perspectives

In this section, issues referring to the current form of our ‘P-D, χ_ε_’ formulation are discussed, including its evolution to a complete concept capable of describing more general categories of materials, in addition to the LHI discussed above, such as non-homogeneous and non-isotropic ones.

A careful assessment of Section 4 reveals that the same argumentation and algebra still hold for the case of a linear and isotropic, however non-homogeneous, material that exhibits a spatially-dependent *intrinsic* electric susceptibility, $\chi_{e}(r)$ and $\chi_{\varepsilon }(r)$, according to the ‘P-E, χ_e_’ and ‘P-D, χ_ε_’ formulations, respectively. Notably, the same holds for the case of a linear and homogeneous, however non-isotropic, material characterized by a spatially-independent, *intrinsic* electric susceptibility tensor, ${\bar{\chi }}_{e}$ and ${\bar{\chi }}_{\varepsilon }$, according to the ‘P-E, χ_e_’ and ‘P-D, χ_ε_’ formulations, respectively [10,11,12,26]. In this case, the respective *intrinsic* relative electric permittivity tensor should read, ${\bar{\varepsilon }}_{r}=\bar{I}+{\bar{\chi }}_{e}$ and ${\bar{\varepsilon }}_{r\varepsilon }={\left(\bar{I}+{\bar{\chi }}_{\varepsilon }\right)}^{-1}$, for the ‘P-E, χ_e_’ and ‘P-D, χ_ε_’ formulation, respectively. Here $\bar{I}$ is the identity tensor (else, unit matrix) and ${\left(\bar{I}+{\bar{\chi }}_{\varepsilon }\right)}^{-1}$ denotes the inverse of the tensor $\left(\bar{I}+{\bar{\chi }}_{\varepsilon }\right)$ [10,11,12,26,34,46]. Simple algebra verifies that in this case, relation (18) should take the form $\left(\bar{I}+{\bar{\chi }}_{\varepsilon }\right)={\left(\bar{I}+{\bar{\chi }}_{e}\right)}^{-1}$, else $\left(\bar{I}+{\bar{\chi }}_{\varepsilon }\right)\left(\bar{I}+{\bar{\chi }}_{e}\right)=\bar{I}$. These relations take the equivalent forms, ${\bar{\chi }}_{\varepsilon }=-{\bar{\chi }}_{e}/(\bar{I}+{\bar{\chi }}_{e})$, else ${\bar{\chi }}_{e}=-{\bar{\chi }}_{\varepsilon }/(\bar{I}+{\bar{\chi }}_{\varepsilon })$, as well. Strikingly, it can be easily verified that the combination of the above two cases holds, as well. Thus, in the more generic case of a linear, non-homogeneous, and non-isotropic material that exhibits both spatial-dependence and orientation-dependence in the electric susceptibility, ${\bar{\chi }}_{e}(r)$ and ${\bar{\chi }}_{\varepsilon }(r)$, the two independent formulations, ‘P-E, χ_e_’ and ‘P-D, χ_ε_’, are equivalent. This rather unexpected generalization from the LHI materials discussed until now in this work stems from the fact that the electric permittivities of the two independent formulations, ‘P-E, χ_e_’ and ‘P-D, χ_ε_’, are equivalent, $\varepsilon =\varepsilon_{\varepsilon }$, whether they are spatially and directionally dependent or not. This is so because the connection of the parent susceptibilities, relation (18), originates from a generic linear fractional transformation, not subjected to any restrictions, having the form [47].

(119) $$X(x)=\frac{ax+b}{cx+d}$$

where x stands for $\chi_{e}$ and $\chi_{\varepsilon }$, when X(x) stands for $\chi_{\varepsilon }$ and $\chi_{e}$, respectively. In our case, all a, b, c, d∈Z, while the transformation is invertible since from relation (18) it is seen that a=−1, b=0, c=1, and d=1, so that $\left(ad-bc\right)=-1$ [47]. This transformation maps the entire positive $\chi_{e}$-axis ($0\leq \chi_{e}<\infty$) to part of the negative $\chi_{\varepsilon }$ axis ($-1\leq \chi_{\varepsilon }\leq 0$) and vice versa.

The facts discussed above strongly upgrade the applicability of the ‘P-D, χ_ε_’ formulation and its authentication as a reliable aspect that is probably absolutely equivalent to the ‘P-E, χ_e_’ one in all cases, even for non-linear, non-homogeneous, and non-isotropic materials. Nevertheless, while the above arguments are rather clearly verified, it should be stressed that a more detailed investigation is needed for the reliable documentation of the absolute equivalence of the ‘P-E, χ_e_’ and ‘P-D, χ_ε_’ formulations. For instance, the above discussion holds when the physical parameters of the material’s polarization, i.e., susceptibility and permittivity, are time-independent. The time-dependent case should be carefully addressed, as well. Also, the case of non-linear dielectric materials should be investigated in detail. These considerations will be the subject of future work.

In addition, except for the relatively simple cases of the external applied field (e.g., uniform and radial) discussed above in Section 2 and Section 3, in recent publications of ours [48,49,50,51], we have considered dielectric and magnetic spheres and cylinders subjected to *any* form of external applied field. In these works, the standard ‘P-E, χ_e_’ formulation was employed, and closed-form, universal relations were obtained for the polarization, electric and magnetic, of the spherical and cylindrical specimens [48,49,50,51]. Importantly, in these works, the depolarizing effect was taken into account explicitly through the shape and dimensions of the specimen, and also provided closed-form relations for the depolarizing factor, $N_{e}$, and the *extrinsic* susceptibility, $\chi_{e,extr}$, of the employed specimen with respect to the *intrinsic* susceptibility, $\chi_{e,intr}=\chi_{e}$, of the parent material. Following the strategy introduced in [48,49,50,51], in future work, the respective results can be deduced for the ‘P-D, χ_ε_’ formulation. This would clearly document the consistency of the ‘P-D, χ_ε_’ formulation with the ‘P-E, χ_e_’ one for the quite complicated cases discussed in [48,49,50,51].

Returning to the findings documented in the present work, it is clearly evidenced that the ‘P-D, χ_ε_’ formulation is at least equivalent, if not advantageous to, the standard ‘P-E, χ_e_’ one for the description of time-dependent processes. Thus, the ‘P-D, χ_ε_’ formulation can be employed for the description of relevant processes in both basic research and applications; in the last decades, dielectric materials have been studied widely in the form of spherical and cylindrical structures. For instance, such structures are used in applications of microwave absorption and shielding/invisibility [52,53,54,55], to model colloidal particles and biological cells during their sorting/manipulation, and to describe the micromanipulation and controlled assembly of colloidal particles [56,57,58,59,60,61,62,63,64]. For detailed information, see [48,49,50,51] and references therein.

## 6. Conclusions

Here, the electric susceptibility and polarization were revisited for the wide class of LHI materials. The ‘P-D, χ_ε_’ formulation ($\tilde{P}$(**r**,t) = −**P**(**r**,t) = χ_ε_**D**(**r**,t) with −1 ≤ χ_ε_ ≤ 0) was introduced for the time-dependent case and investigated in comparison to the ‘P-E, χ_e_’ formulation of electricity (**P**(**r**,t) = χ_e_ε_0_**E**(**r**,t) with 0 ≤ χ_e_ < ∞) and the ‘M-H, χ_m_’ formulation of magnetism (**M**(**r**,t) = χ_m_**H**(**r**,t) with −1 ≤ χ_m_ < ∞). The depolarizing effect was taken into account, and a series scheme was introduced to describe the underlying physics of the polarization, and the *extrinsic* susceptibility of the specimen was calculated from the *intrinsic* susceptibility of the parent material. It was shown that in realistic specimens, subjected to the depolarizing effect, the ‘P-E, χ_e_’ formulation suffers from an inherent non-compliance with the accompanying convergence conditions. On the other hand, both the ‘P-D, χ_ε_’ formulation of electricity and the ‘M-H, χ_m_’ formulation of magnetism comply with these demands in all instances. Referring to electricity, the comparison of the ‘P-E, χ_e_’ and ‘P-D, χ_ε_’ formulations revealed, first, their absolute equivalence in the assessment of realistic systems, including the depolarizing effect and time-dependence, and second, the superiority of the ‘P-D, χ_ε_’ formulation in the restoration of the conceptual and mathematical flaws which are inherent in the ‘P-E, χ_e_’ one. The latter were cloaked until now by the fact that in all fundamental relations of electromagnetism, the susceptibility, χ_e_, does not appear as a standalone physical parameter; only the electric permittivity appears, which is the same, $\varepsilon =\varepsilon_{\varepsilon }$, in both the ‘P-E, χ_e_’ and the ‘P-D, χ_ε_’ notations. Last, but not least, it was proved that the ‘P-D, χ_ε_’ formulation does not alter Maxwell’s equations. Accordingly, instead of the standard ‘P-E, χ_e_’ formulation, the ‘P-D, χ_ε_’ one can be employed equally well to describe consistently all time-dependent processes of electromagnetic theory in LHI materials.

## Figures and Tables

**Table 1:** (Left column) Total electric field, $E^{in}\left(r,t\right)$, *internal* electric field, $E_{int}^{in}\left(r,t\right)$, and polarization, $P\left(r,t\right)$, at the interior of a specimen with depolarizing factor, N, subjected to an *external* electric field, $E_{ext}\left(r,t\right)=E_{0}f\left(\omega t\right)\hat{z}$, according to the ‘P-E, χ_e_’ formulation. Also, *bound* charge surface density, $\sigma_{b}\left(r,t\right){|}_{S}$, residing at the specimen/vacuum interface. (Right column) Total electric displacement, $D_{in}\left(r,t\right)$, *internal* electric displacement, $D_{int}^{in}\left(r,t\right)$, and reverse polarization, $\tilde{P}\left(r,t\right)=-P\left(r,t\right)$, at the interior of a specimen with depolarizing factor, N, subjected to an *external* electric displacement, $D_{ext}\left(r,t\right)=D_{0}f\left(\omega t\right)\hat{z}$, according to the ‘P-D, χ_ε_’ formulation. Also, reverse *bound* charge surface density, ${\tilde{\sigma }}_{b}\left(r,t\right){|}_{S}=-\sigma_{b}\left(r,t\right){|}_{S}$, residing at the specimen/vacuum interface.

‘P-E, χ_e_’ Formulation	‘P-D, χ_ε_’ Formulation
$E^{in}\left(r,t\right)=\left(\frac{1}{1+N\chi_{e}}\right)E_{ext}\left(r,t\right)$	$D^{in}\left(r,t\right)=\left(\frac{1}{1+N\chi_{\varepsilon }}\right)D_{ext}\left(r,t\right)$
$E_{int}^{in}\left(r,t\right)=-\left(\frac{N\chi_{e}}{1+N\chi_{e}}\right)E_{ext}\left(r,t\right)$	$D_{int}^{in}\left(r,t\right)=-\left(\frac{N\chi_{\varepsilon }}{1+N\chi_{\varepsilon }}\right)D_{ext}\left(r,t\right)$
$P\left(r,t\right)=\left(\frac{\chi_{e}}{1+N\chi_{e}}\right)\left(\varepsilon_{0}E_{ext}\left(r,t\right)\right)$	$\tilde{P}\left(r,t\right)=\left(\frac{\chi_{\varepsilon }}{1+N\chi_{\varepsilon }}\right)D_{ext}\left(r,t\right)$
$\sigma_{b}\left(r,t\right){|}_{S}=\left(\frac{\chi_{e}}{1+N\chi_{e}}\right)\hat{n}\cdot \left(\varepsilon_{0}E_{ext}\left(r,t\right)\right){|}_{S}$	${\tilde{\sigma }}_{b}\left(r,t\right){|}_{S}=\left(\frac{\chi_{\varepsilon }}{1+N\chi_{\varepsilon }}\right)\;\hat{n}\cdot D_{ext}\left(r,t\right){|}_{S}$

**Table 2:** (Left column) System of a dielectric specimen originating from an LHI parent material of *intrinsic* electric susceptibility $\chi_{e}$ and $\chi_{\varepsilon }$, according to the ‘P-E’ and ‘P-D’ formulation, respectively. The specimen is subjected to a static, *external* electric field originating from *free* charges. (Middle column) The polarization, $P\left(r\right)$, at the interior of the specimen having depolarizing factor, $N_{e}$, according to the ‘P-E, χ_e_’ formulation. Two versions of $P\left(r\right)$ are provided in all cases, the as-obtained (top of the cell) and a reformed (bottom of the cell). (Right column) The reverse polarization, $\tilde{P}\left(r\right)$, at the interior of the specimen having depolarizing factor, $N_{\varepsilon }$, according to the ‘P-D, χ_ε_’ formulation. Two versions of $\tilde{P}\left(r\right)$ are provided in all cases, the as-obtained (top of the cell) and a reformed (bottom of the cell). The as-obtained versions of $P\left(r\right)$ and $\tilde{P}\left(r\right)$ are reproduced from [21]. The reformed versions of $P\left(r\right)$ and $\tilde{P}\left(r\right)$, introduced in the present work, highlight the presence of the depolarizing factors $N_{e}$ (middle column) and $N_{\varepsilon }$ (right column); it becomes clear that in all pairs, $N_{e}+N_{\varepsilon }=1$.

System	$\mathrm{Polarization},\;P\left(r\right)$ ‘P-E, χ_e_’ Formulation	$\mathrm{Reverse}\;\mathrm{Polarization},\;\tilde{P}\left(r\right)$ ‘P-D, χ_ε_’ Formulation
Dielectric LHI sphere with P-E/P-D electric susceptibility $\chi_{e}/\chi_{\varepsilon }$ and radius a, subjected to an *external*, uniform electric field/displacement field directed along the *z*-axis, $E\left(r\right)=E_{0}\hat{z}/D\left(r\right)=D_{0}\hat{z}$.	$P\left(r\right)=\left(\frac{3\chi_{e}}{\varepsilon_{r}+2}\right)\varepsilon_{0}E_{0}\hat{z}$ else $P\left(r\right)=\left(\frac{\chi_{e}}{1+N_{e}\chi_{e}}\right)\varepsilon_{0}E_{0}\hat{z}$ where $N_{e}=1/3$	$\tilde{P}\left(r\right)=-P\left(r\right)=\left(\frac{3\chi_{\varepsilon }}{2\chi_{\varepsilon }+3}\right)D_{0}\hat{z}$ else $\tilde{P}\left(r\right)=-P\left(r\right)=\left(\frac{\chi_{\varepsilon }}{1+N_{\varepsilon }\chi_{\varepsilon }}\right)D_{0}\hat{z}$ where $N_{\varepsilon }=2/3$ and $D_{0}=\varepsilon_{0}E_{0}$
Dielectric LHI sphere with P-E/P-D electric susceptibility $\chi_{e}/\chi_{\varepsilon }$ and radius a, with a point electric charge, Q, at its center.	$P\left(r\right)=\left(\frac{\chi_{e}}{\varepsilon_{r}}\right)\left(\frac{Q}{4\pi r^{2}}\right)\hat{r}$ else $P\left(r\right)=\left(\frac{\chi_{e}}{1+N_{e}\chi_{e}}\right)\left(\frac{Q}{4\pi r^{2}}\right)\hat{r}$ where $N_{e}=1$	$\tilde{P}\left(r\right)=-P\left(r\right)=\chi_{\varepsilon }\left(\frac{Q}{4\pi r^{2}}\right)\hat{r}$ else $\tilde{P}\left(r\right)=-P\left(r\right)=\left(\frac{\chi_{\varepsilon }}{1+N_{\varepsilon }\chi_{\varepsilon }}\right)\left(\frac{Q}{4\pi r^{2}}\right)\hat{r}$ where $N_{\varepsilon }=0$
Dielectric LHI cylinder with P-E/P-D electric susceptibility $\chi_{e}/\chi_{\varepsilon }$, radius a, and infinite length, subjected to an *external*, uniform electric field/displacement field along the *x*-axis, $E\left(r\right)=E_{0}\hat{x}/D\left(r\right)=D_{0}\hat{x}$.	$P\left(r\right)=\left(\frac{2\chi_{e}}{\varepsilon_{r}+1}\right)\varepsilon_{0}E_{0}\hat{x}$ else $P\left(r\right)=\left(\frac{\chi_{e}}{1+N_{e}\chi_{e}}\right)\varepsilon_{0}E_{0}\hat{x}$ where $N_{e}=1/2$	$\tilde{P}\left(r\right)=-P\left(r\right)=\left(\frac{2\chi_{\varepsilon }}{\chi_{\varepsilon }+2}\right)D_{0}\hat{x}$ else $\tilde{P}\left(r\right)=-P\left(r\right)=\left(\frac{\chi_{\varepsilon }}{1+N_{\varepsilon }\chi_{\varepsilon }}\right)D_{0}\hat{x}$ where $N_{\varepsilon }=1/2$ and $D_{0}=\varepsilon_{0}E_{0}$
Dielectric LHI cylinder with P-E/P-D electric susceptibility $\chi_{e}/\chi_{\varepsilon }$, radius a, and infinite length, with a coaxial, homogeneous, linear charge density, $\lambda_{0}$.	$P\left(r\right)=\left(\frac{\chi_{e}}{\varepsilon_{r}}\right)\left(\frac{\lambda_{0}}{2\pi \rho }\right)\hat{\rho }$ else $P\left(r\right)=\left(\frac{\chi_{e}}{1+N_{e}\chi_{e}}\right)\left(\frac{\lambda_{0}}{2\pi \rho }\right)\hat{\rho }$ where $N_{e}=1$	$\tilde{P}\left(r\right)=-P\left(r\right)=\chi_{\varepsilon }\left(\frac{\lambda_{0}}{2\pi \rho }\right)\hat{\rho }$ else $\tilde{P}\left(r\right)=-P\left(r\right)=\left(\frac{\chi_{\varepsilon }}{1+N_{\varepsilon }\chi_{\varepsilon }}\right)\left(\frac{\lambda_{0}}{2\pi \rho }\right)\hat{\rho }$ where $N_{\varepsilon }=0$
Dielectric LHI slab with P-E/P-D electric susceptibility $\chi_{e}/\chi_{\varepsilon }$, thickness a, and infinite length, subjected to an *external*, uniform electric field/displacement field, $E\left(r\right)=E_{0}\hat{z}/D\left(r\right)=D_{0}\hat{z}$.	$P\left(r\right)=\left(\frac{\chi_{e}}{\varepsilon_{r}}\right)\varepsilon_{0}E_{0}\hat{z}$ else $P\left(r\right)=\left(\frac{\chi_{e}}{1+N_{e}\chi_{e}}\right)\varepsilon_{0}E_{0}\hat{z}$ where $N_{e}=1$	$\tilde{P}\left(r\right)=-P\left(r\right)=\chi_{\varepsilon }D_{0}\hat{z}$ else $\tilde{P}\left(r\right)=-P\left(r\right)=\left(\frac{\chi_{\varepsilon }}{1+N_{\varepsilon }\chi_{\varepsilon }}\right)D_{0}\hat{z}$ where $N_{\varepsilon }=0$ and $D_{0}=\varepsilon_{0}E_{0}$

## Data Availability

The original contributions presented in this study are included in the article. Further inquiries can be directed to the author.

## Appendix A

In this appendix, the basics of the electric polarization, i.e., electric susceptibility/relative permittivity, are discussed, while typical values of the relative permittivity in representative substances are recalled. Briefly, in LHI dielectrics, the polarization originates from the electronic (optical), ionic (atomic), and dipolar (orientational) polarizabilities [35,36,37,38,39,40,41].

Electronic polarization stems from the spatial redistribution of the electrons surrounding the neutral atoms of a molecule or a crystal under the application of an external electric field. In the static (low-frequency) case, this process results in an electronic polarizability $\alpha_{e}={\left(Zq\right)}^{2}/m\omega_{o}^{2}$, where Z is the number of electrons (atomic number), q is the electron charge, m is the electron mass, and $\omega_{o}={\left(K/m\right)}^{1/2}$ is the resonance frequency, with $K={\left(Zq\right)}^{2}/4\pi \varepsilon_{0}R^{3}$ an effective constant of the restoring force and *R* an effective radius of the electron cloud [35,36,37,38,39,40,41]. The electric susceptibility of electronic origin is $\chi_{e,e}=(N_{e}/\varepsilon_{0})\alpha_{e}=(N_{e}/\varepsilon_{0})({\left(Zq\right)}^{2}/m\omega_{o}^{2})$, where $N_{e}$ is the number of induced dipoles per unit volume.

Ionic polarization originates from the relative displacement between the positively and negatively charged atoms of an ionic molecule or crystal. For the toy model of a linear diatomic structure of atoms A (mass $m_{A}$, charge $q_{A}=Zq$) and B (mass $m_{B}$, charge $q_{B}=-q_{A}$) with interatomic distance a, the static (low frequency) ionic polarizability is $\alpha_{i}={\left(Zq\right)}^{2}/m_{r}\omega_{o}^{2}$, where Z is the ionic number of the atoms, q is the electron charge, $m_{r}=m_{A}m_{B}/(m_{A}+m_{B})$ is the reduced mass of atoms A and B, and $\omega_{o}={\left({2K}_{i}/m_{r}\right)}^{1/2}$ is the resonance frequency with $K_{i}=2{\left(Zq\right)}^{2}/4\pi \varepsilon_{0}a^{3}$ an effective constant of the restoring force [35,36,37,38,39,40,41]. The electric susceptibility of ionic origin is $\chi_{e,i}=(N_{i}/\varepsilon_{0})\alpha_{i}=(N_{i}/\varepsilon_{0})({\left(Zq\right)}^{2}/m_{r}\omega_{o}^{2})$, where $N_{i}$ is the number of induced dipoles per unit volume.

Dipolar polarization is inherent to some molecules due to the endogenous heterogeneous distribution of charges in their building units, the atoms. Accordingly, these molecules are called polar and exhibit a permanent electric dipole moment which aligns to an external electric field. The following results are based on the method introduced by Langevin in the study of paramagnetic materials, which was later adopted by Debye to address the respective processes in electricity. The static (low-frequency) dipolar polarizability is $\alpha_{d}=p^{2}/3k_{B}T$, where p is the moment of each permanent electric dipole, $k_{B}$ is the Boltzmann constant, and T is the temperature [35,36,37,38,39,40,41]. The electric susceptibility of dipolar origin is $\chi_{e,d}=(N_{d}/\varepsilon_{0})\alpha_{d}=(N_{d}/\varepsilon_{0})(p^{2}/3k_{B}T)$, where $N_{d}$ is the number of permanent dipoles per unit volume that are available to align with the electric field.

This basic knowledge, based on the ‘P-E, χ_e_’ formulation, predicts quite efficiently the electric susceptibility/relative permittivity, $\chi_{e}$/$\varepsilon_{r}$, of many materials. Table A1 reproduces some representative cases for the static relative permittivity, $\varepsilon_{r}$, obtained at room temperature and ambient pressure (except when mentioned differently) [38,39,40,41].

**Table A1:** Relative permittivity, $\varepsilon_{r}$, of some representative substances.

Substance	Phase	$\mathrm{Relative}\;\mathrm{Permittivity},\;\varepsilon_{r}$
Helium	Gas	1.000065
Neon	Gas	1.00013
Hydrogen	Gas	1.00024
Argon	Gas	1.000517
Nitrogen	Gas	1.000548
Carbon dioxide	Gas	1.00092
Water (at T = 100 °C)	Gas	1.00589
Argon (at T < 87.3 K)	Liquid	1.53
Chloroform	Liquid	4.78
Methanol	Liquid	33.6
Silicone oil	Liquid	2.5
Water	Liquid	81
Water (at T = –30 ^ο^C)	Solid	104
Paper	Solid	3.85
Teflon	Solid	2.1
Nylon	Solid	3.4
PVC (polyvinyl chloride)	Solid	4.6
PS (polystyrene)	Solid	2.6
NaCl	Solid	5.9
KCl	Solid	4.84
KBr	Solid	4.9
Al_2_O_3_	Solid	8.5
MgO	Solid	9.8
Si	Solid	11.9

## Appendix B

In this appendix, the basics of the magnetic polarization, i.e., magnetic susceptibility/relative permittivity, are discussed, while typical values of the relative permittivity in representative substances are recalled [26,41,42].

Referring to diamagnetism, the Langevin type has $\chi_{m}=-\left(\mu_{0}N_{a}Ze^{2}/6m\right)\langle r^{2}\rangle$, while the Landau type has $\chi_{m}=-\left(\mu_{0}e^{2}/12\pi^{2}m\hbar \right)\sqrt{2mE_{F}}$. Regarding paramagnetism, for the general Curie type, at relatively low magnetic fields, the following relation holds $\chi_{m}=N_{a}g_{J}^{2}J(J+1)\mu_{B}^{2}/3k_{B}T$, while for the Pauli type, we have $\chi_{m}=\mu_{0}\mu_{B}^{2}g\left(E_{F}\right)$. In the above relations, $\mu_{0}$ is the permeability of vacuum, Z the atomic number, $N_{a}$ is the number of atoms per unit volume, e and m are the charge and mass of the electron, $\langle r^{2}\rangle$ is the mean square distance of the electrons from the nucleus, ħ is the reduced Plank constant, $E_{F}$ is the Fermi energy, $g_{J}$ is the Landé g-factor, J is the angular momentum, $\mu_{B}$ is the Bohr magneton, $k_{B}$ is the Boltzmann constant, and $g(E_{F})$ is the electronic density of states at the Fermi energy [26,41,42].

These standard approaches predict quite effectively the magnetic susceptibility/relative permeability, $\chi_{m}$/$\mu_{r}$, of many materials. Table A2 summarizes representative cases of substances obtained at room temperature and ambient pressure.

**Table A2:** Relative permeability, $\mu_{r}$, and respective magnetic susceptibility, $\chi_{m}$, of some representative diamagnetic and paramagnetic substances.

Substance	$\mathrm{Relative}\;\mathrm{Permeability},\;\mu_{r}$	$\mathrm{Magnetic}\;\mathrm{Susceptibility},\;\chi_{m}$
Gold	0.999964	−0.000036
Silver	0.9999736	−0.0000264
Lead	0.9999831	−0.0000169
Copper	0.9999906	−0.0000094
Water	0.999992	−0.000008
Silicon	0.9999958	−0.0000042
Teflon	1	0
Hydrogen	1	0
Calcium	1.000019	0.000019
Oxygen	1.000002	0.000002
Aluminum	1.000021	0.000021
Chromium	1.00027	0.00027
Platinum	1.0003	0.0003
Palladium	1.0008	0.0008
Manganese	1.001	0.001
Austenitic stainless steel	1.003–1.05	0.003–0.05
